# Nitrogen cycle microorganisms can be reactivated after Space exposure

**DOI:** 10.1038/s41598-018-32055-4

**Published:** 2018-09-13

**Authors:** Ralph E. F. Lindeboom, Chiara Ilgrande, José M. Carvajal-Arroyo, Ilse Coninx, Olivier Van Hoey, Hugo Roume, Julia Morozova, Kai M. Udert, Benedikt Sas, Christel Paille, Christophe Lasseur, Vyacheslav Ilyin, Peter Clauwaert, Natalie Leys, Siegfried E. Vlaeminck

**Affiliations:** 1 Center for Microbial Ecology and Technology (CMET), Ghent University, Coupure Links 653, 9000 Gent, Belgium; 2 Section Sanitary Engineering, Department of Water Management, Faculty of Civil Engineering and Geosciences, Delft University of Technology, Stevinweg 1, 2628CN Delft, The Netherlands; 3 Unit of Microbiology, Belgian Nuclear Research Centre (SCK•CEN), Boeretang 200, 2400 Mol, Belgium; 4 Unit of Research in Dosimetric Applications, Belgian Nuclear Research Centre (SCK•CEN), Boeretang 200, 2400 Mol, Belgium; 5 MetaGenoPolis, INRA, Université Paris-Saclay Domaine de Vilvert, Bat. 325 78352, Jouy-en-Josas, France; 6 Institute of Biomedical Problems (IMBP), State Research Center of The Russian Federation, Khoroshevskoye Shosse, 76a, 123007 Moscow, Russia; 7 Eawag, Swiss Federal Institute of Aquatic Science and Technology, 8600, Dübendorf, Switzerland; 8 Laboratory of Food Microbiology and Food Preservation, Ghent University, Coupure links 653, 9000 Gent, Belgium; 9 ESA/ESTEC Keplerlaan 1, 2201 AZ Noordwijk, The Netherlands; 10 Research of Sustainable Energy, Air and Water Technology, Department of Bioscience Engineering, University of Antwerp, Groenenborgerlaan 171, 2020 Antwerpen, Belgium; 11 ETH Zürich, Institute of Environmental Engineering, 8093 Zürich, Switzerland

## Abstract

Long-term human Space missions depend on regenerative life support systems (RLSS) to produce food, water and oxygen from waste and metabolic products. Microbial biotechnology is efficient for nitrogen conversion, with nitrate or nitrogen gas as desirable products. A prerequisite to bioreactor operation in Space is the feasibility to reactivate cells exposed to microgravity and radiation. In this study, microorganisms capable of essential nitrogen cycle conversions were sent on a 44-days FOTON-M4 flight to Low Earth Orbit (LEO) and exposed to 10^−3^–10^−4^ g (gravitational constant) and 687 ± 170 µGy (Gray) d^−1^ (20 ± 4 °C), about the double of the radiation prevailing in the International Space Station (ISS). After return to Earth, axenic cultures, defined and reactor communities of ureolytic bacteria, ammonia oxidizing archaea and bacteria, nitrite oxidizing bacteria, denitrifiers and anammox bacteria could all be reactivated. Space exposure generally yielded similar or even higher nitrogen conversion rates as terrestrial preservation at a similar temperature, while terrestrial storage at 4 °C mostly resulted in the highest rates. Refrigerated Space exposure is proposed as a strategy to maximize the reactivation potential. For the first time, the combined potential of ureolysis, nitritation, nitratation, denitrification (nitrate reducing activity) and anammox is demonstrated as key enabler for resource recovery in human Space exploration.

## Introduction

Long-term human Space missions or habitation require resource efficient processes to produce food, water and oxygen from wastes, in so-called regenerative life support systems (RLSS) to be independent from terrestrial resupply^1^. For efficient nitrogen conversion, microbial biotechnology is the preferred strategy^2^. Starting mainly from organic nitrogen, nitrate (NO_3_^−^) or nitrogen gas (N_2_) can be recovered over ammoniacal nitrogen (NH_3_/NH_4_^+^) and nitrite (NO_2_^−^). Key processes therefore include ureolysis, nitrification, denitrification and anammox, performed respectively by ureolytic bacteria, ammonia oxidizing bacteria (AOB) and archaea (AOA), nitrite oxidizing bacteria (NOB), denitrifying bacteria and anaerobic ammonium oxidizing bacteria (anammox). In MELiSSA, the European Space Agency’s Micro-Ecological Life Support System Alternative for example, nitrogen recovery is foreseen through nitrification, with *Nitrosomonas europaea* as AOB and *Nitrobacter winogradskyi* as NOB^3^. Short-term nitrification activity has been observed in an aquarium in Space^4^, but long term studies on the effect of micro-gravity and radiation are still in preparation^2^.

A prerequisite to long-term bioreactor operation in Space is the ability to preserve microorganisms, to enable a fast system start-up and recovery after process failure, which could take months due to the slow growth of autotrophs^5,6^. Under terrestrial storage conditions, it was demonstrated in previous studies that AOB and anammox activity could be recovered after 5 months at 4 and 20 °C^7^, while long-term storage procedures needed further optimization^8,9^. Functional resilience and resistance are influenced by the diversity of the microbial community^10^ and, in Space, the exposure to microgravity and radiation may additionally affect stored microorganisms^11^.

**Figure 1:**
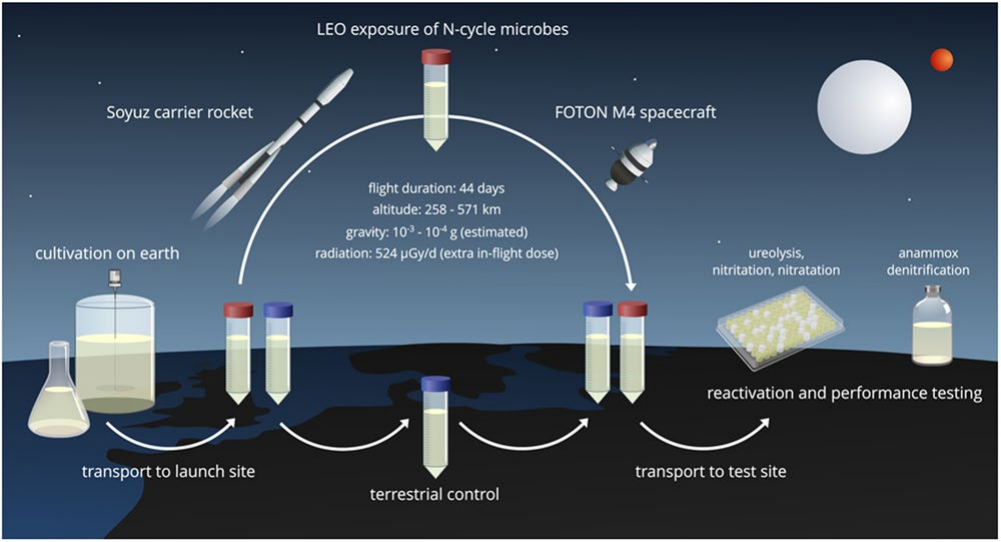
Overview of the experimental steps of the LEO exposure and reactivation set-up.

In this study, a broad spectrum of nitrogen cycle microorganisms was brought for 44 days into Low Earth Orbit (LEO), a geocentric orbit with altitudes ranging from 160 to 2000 km^12^, serving as a proxy for exposure to Space conditions (Fig. 1). The test included three axenic cultures, two defined communities and three reactor communities, capable of ureolysis, nitrification, full denitrification and/or anammox. For some conversions (e.g. anammox) and types of organisms (e.g. AOA), this is the first report on the effect of Space exposure. It was hypothesized that after storage (1) LEO flight cultures (F, around 20 °C) would have a lower activity than cultures stored on ground at a similar temperature (G23, stored at 23 °C) (effect of Space: F < G23), (2) terrestrial storage at lower temperature (G4, refrigerated at 4 °C) would have the best reactivation potential (effect of temperature: G4 > G23), and (3) cultures with a higher microbial and therefore metabolic diversity would be more resilient to preservation.

## Results and Discussion

### Microbial characterization of the cultures

For axenic cultures and defined autotrophic communities, heterotrophic contamination was evaluated via colony count on LB agar plates. Contaminated samples (S1.2) were discarded from the activity test from a planetary defense perspective.

The reactor communities were phylogenetically characterized with Illumina after LEO exposure, with details on the methods and results described in Supporting Information S1.3 and S3, respectively. Eveness (Pielou) was rather similar for all three, while diversity (Inverse Simpson) was highest for Nitr-au, a urea- and nitrite-fed nitrification suspension, and OLAND, an ammonium-fed partial nitritation/anammox biofilm, and lower for Nitr-ur, urine-fed nitrification biofilm (Table 1).

**Table 1:** Overview of characteristics of the cultures.

Culture abbreviation	C	Ns	Nb	NsNb	CNsNb	Nitr-ur	Nitr-au	OLAND
Ureolytic member(s)	*Cupriavidus pinatubonensis* strain 1245 T	—	—		*Cupriavidus pinatubonensis* strain 1245 T	Not known	Not known	Not known
AOB/AOA member(s)	—	*Nitrosomonas europaea* strain Winogradsky	—	*Nitrosomonas europaea* strain Winogradsky	*Nitrosomonas europaea* strain Winogradsky	*Nitrosomonas sp*.	Thaumarchaeota	*Nitrosomonas sp*.
NOB member(s)	—	—	*Nitrobacter winogradskyi* strain Nb_255	*Nitrobacter winogradskyi* strain Nb_255	*Nitrobacter winogradskyi* strain Nb_255	*Nitrobacter winogradskyi*	*Nitrobacter winogradskyi* > *Nitrospira spp*.	*Nitrospira sp. > Nitrobacter winogradskyi*
Denitrifying member(s)	*Cupriavidus pinatubonensis* strain 1245 T^*^	—	—	—	*Cupriavidus pinatubonensis* strain 1245 T^*^	Not known	Not known	Not known
Anammox member(s)	—	—	—	—	—	—	—	*Ca. Kuenenia* ^14^
Medium^#^	MM284gluc	ATCC 2265 Nitrosomonas europaea	DMSZ 756c Autotrophic Nitrobacter	Mixture Ns & Nb	Mixture C, Ns & Nb	Partially nitrified real urine (NO_3_^−^N/NH_4_^+^-N ≈ 1)	Synthetic – Urea & nitrite	Synthetic – Ammonium
Protein concentration (g_prot_ L^−1^)	—	0.008	0.019	0.023	0.027	0.532	0.571	0.181
Eveness (Pielou)	0^	0^	0^	0.222^	0.639^	0.562	0.647	0.662
Diversity (Simpson^−1^)	1^	1^	1^	1.3^	2.3^	10	24	25
Source	Belgian Nuclear Research Centre (SCK•CEN), Mol (BE)					Eawag (CH)	ABIL, Avecom, Wondelgem (BE)	CMET, Ghent University (BE)

AOB: ammonia oxidizing bacteria; NOB: nitrite oxidizing bacteria; anammox: anaerobic ammonium oxidizing bacteria; OLAND: oxygen-limited autotrophic nitrification/denitrification.

^*^C. pinatubonensis strain 1245^T^ can reduce nitrate to nitrite, but does not reduce nitrite to nitrogen gas^23^.

^#^More details are presented in S1.

^Theoretically derived from the composition of the cultures.

In Nitr-ur, *Nitrosomonas sp*. was detected as AOB, and *Nitrobacter winogradskyi* as NOB. In previous studies, the *Nitrosomonas europaea/Nitrosococcus mobilis* lineage was dominant for AOB, containing mainly *Nitrosomonas eutropha*^13^. In Nitr-au, no AOB were found, yet AOA, i.e. members of the phylum *Thaumarchaeota*, appeared. The archaeal OTU0001 was 96% identical to *Nitrososphaera* sp. (Fig. S3.3). As for NOB, mainly *Nitrobacter winogradskyi* was detected, with an OTU count number 23 times higher than that of *Nitrospira spp*. The coexistence of Thaumarcheota, which require low substrate concentrations, and Nitrobacter, which requires high substrate concentrations, can be explained by the composition of the influent utilized in the reactor, a mix of urea and nitrite (Table 1). In OLAND, *Nitrosomonas sp*. was detected as AOB, the same OTU as in Nitr-ur, and for NOB, *Nitrospira sp*. OTU counts outnumbered *Nitrobacter winogradksyi* with a factor 14. The retrieved anammox were OTU0017 and OTU0021, members of the *Brocadiaceae* family, showing 82% similarity to “Candidatus *Kuenenia stuttgartiensis”* (Fig. S3.4). Also for OLAND, detected genus and family levels are similar to previous reports^14^.

### Storage conditions

**Figure 2:**
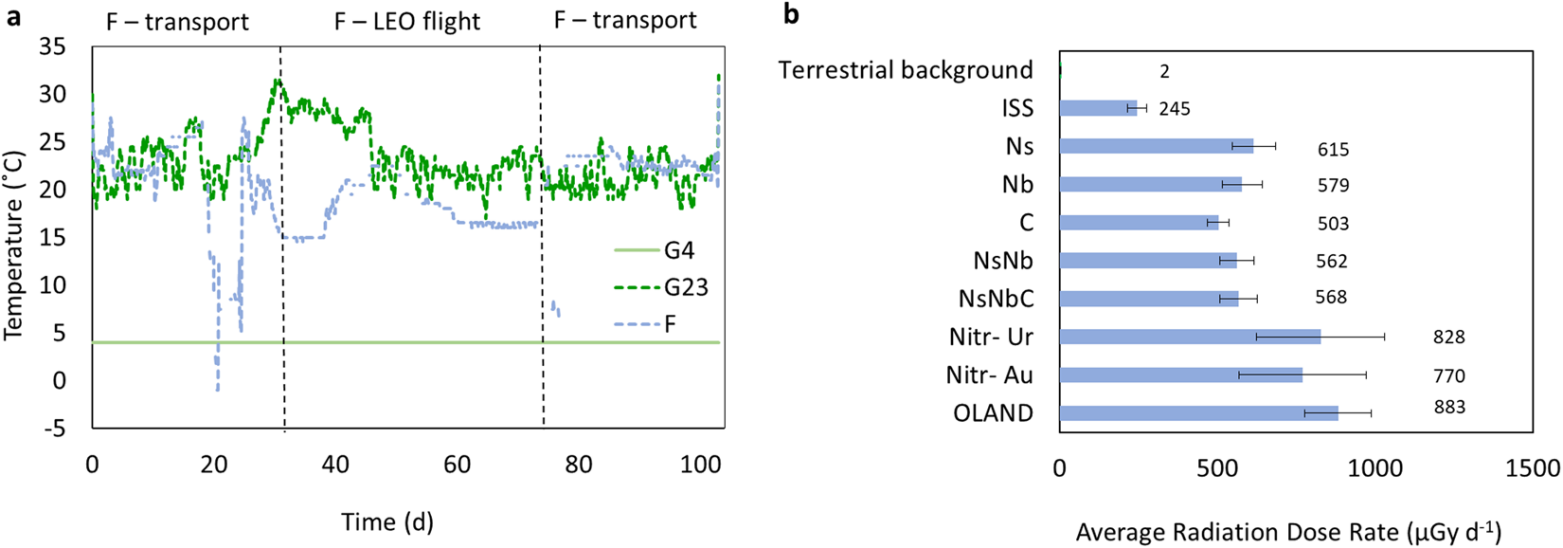
(**a**) Temperature profiles experienced by the cultures preserved on ground at 23 °C (G23), at 4 °C (G4) and in low Earth orbit (LEO) flight (F). (**b**) Radiation dose rates on Earth (terrestrial background measurement assessed in Mol, Belgium), in the International Space Station (ISS; DOSIS-3D, unpublished data SCK•CEN), and experienced by the different cultures during LEO flight (for culture labels, refer to Table 1).

The two ground control groups were exposed to a background radiation of 2.1 ± 0.3 µGy d^-1^ and an ambient temperature of 23.1 ± 3 °C (G23) or a refrigerated temperature of 4 ± 1 °C (G4). The LEO flight conditions exposed the F cultures to 17.5 ± 2 °C, between 10^-3^ and 10^-4^ g (except during launch/landing), and 687 ± 170 µGy d^−1^, more than double the dose experienced in the International Space Station (ISS) (Fig. 2). Including the transport periods, temperature experienced by the F cultures was 19.6 ± 4 °C. The radiation doses recorded differ significantly among tubes (Fig. 2b). This variability is likely due to the limited shielding of the FOTON-M4 satellite against radiation, and hence the location inside the satellite affecting the exact dosage experienced.

### Nitrogen conversion activities after preservation

**Figure 3:**
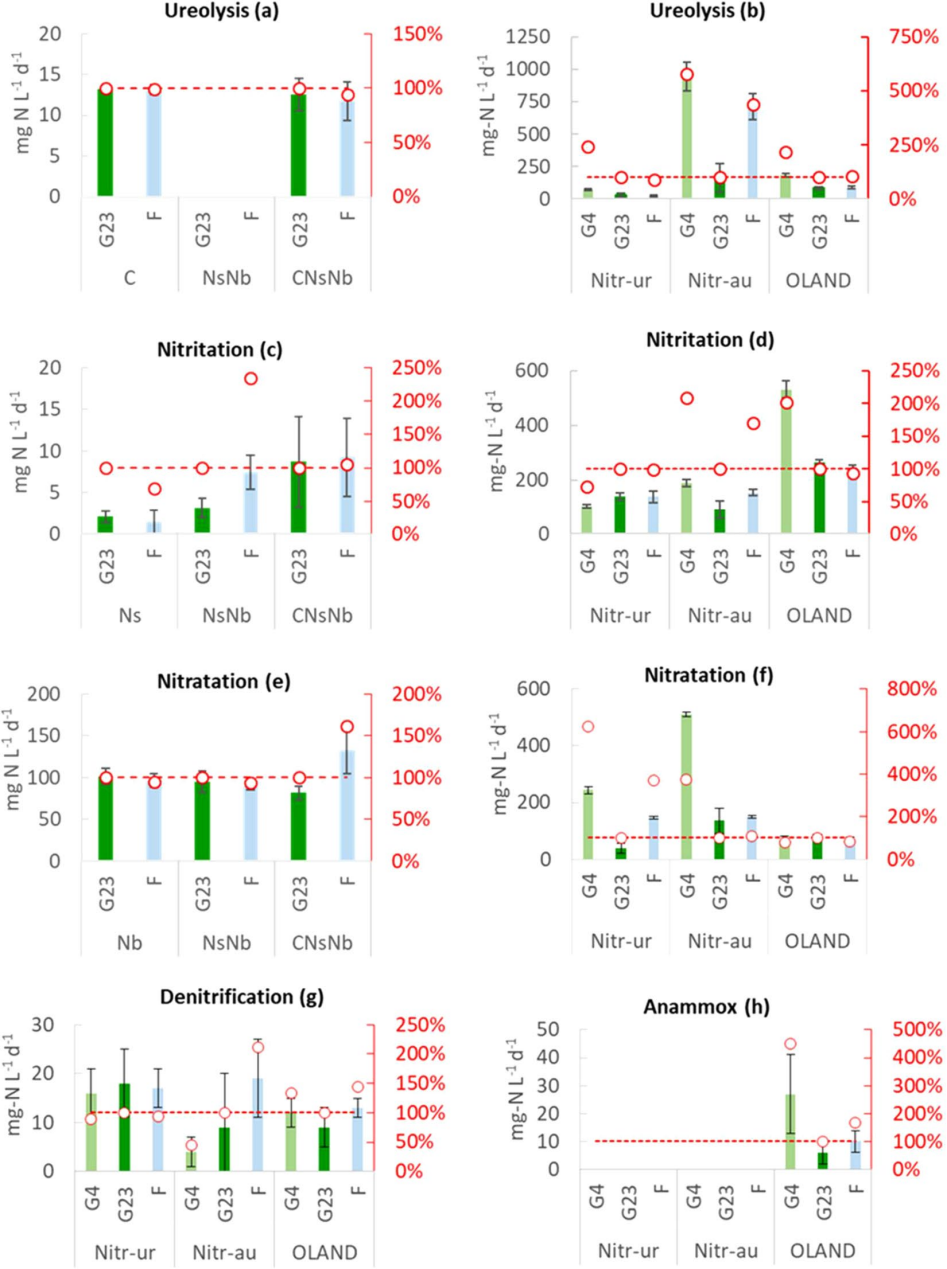
Volumetric activity rates (primary Y axis) for ureolysis (**a**,**b;** ammonium production in presence of 250 mg-N L^−1^ of allylthiourea), nitritation (**c**,**d;** ammonium consumption), nitratation (**e**,**f**; nitrite consumption), denitrification (**g;** nitrite accumulation was not tested) and anammox (**h**; ammonium consumption) for cultures preserved on ground at 4 °C (G4), on ground at 23 °C (G23) and in low Earth orbit flight (F). The relative volumetric (ο) activity (secondary Y axis) is expressed as a percentage of the ground storage activity (G23). The absence of bars in panels a and h indicates that the tests were not performed, as the presence of the specific metabolic activity was not expected, based on the microbial composition of the community.

The activity tests showed that, despite of exposure to rather harsh conditions in Space, all functionalities (ureolysis, nitritation, nitratation, nitrate removal as indicator for denitrification and anammox) could be retrieved at a reasonable rate (Fig. 3). The discussion of each functionality address the hypotheses previously formulated, namely: (1) the exposure to LEO flight conditions will result in lower rates for the exposed cultures (F) compared to cultures stored on ground at a similar temperature (G23), (2) terrestrially refrigerated samples (G4) would have better reactivation potential then G23, and (3) cultures with higher diversity would be more resilient to preservation.

Supporting Information S1.5 and S1.6 presents the reactivation and activity measurement methods. An overview of the volumetric and biomass-specific rates is presented in Tables S4.1, S4.2 and Fig. S4.1, while the nitrogen concentration profiles are presented in Supporting Information S5.

#### Ureolysis

Against hypotheses 1 and 3, neither LEO (F) vs. ground (G23) conditions nor the presence of other bacteria (i.e. the nitrifiers *Nitrosomonas europaea* and *Nitrobacter winogradskyi)* significantly affected the ureolytic activity of *Cupriavidus pinatubonensis* (p > 0.1) (Fig. 3a).

The data from the reactor communities also oppose hypothesis 1, with the ureolytic activity not significantly (p > 0.1) lowered by LEO exposure compared to treatment G23 and even 6 times higher for Nitr-au (p < 0.001), corresponding to the 600% value depicted in Fig. 3b. In agreement with hypothesis 2, refrigerated storage (G4) yielded faster ureolysis than G23 for Nitr-ur (p < 0.05) and OLAND (p < 0.001). Nitr-au seemingly presented faster ureolytic rates in G4 samples, yet this difference is not significant (p > 0.1). The higher diversity shared by Nitr-au and OLAND in contrast to Nitr-ur did not yield a discriminating trend, so hypothesis 3 could not be confirmed.

#### Nitritation

LEO conditions did not significantly lower the aerobic ammonia oxidation rates of *Nitrosomonas europaea*, regardless of the presence of other bacteria (p > 0.1) (Fig. 3c), contradicting hypothesis 1. Interestingly, *Nitrobacter winogradskyi* (NsNb) and *Cupriavidus pinatubonensis* (CNsNb) seemed to positively influence *N. europaea*. Nitritation rates for this defined community were significantly higher (p < 0.001) than for *Nitrosomonas europaea* (Ns) (Fig. 3c), even though the latter contained 1.6 times more *Nitrosomonas* cells (based on culture preparation). This indicates that the presence of other strains had a positive effect on the reactivation of *Nitrosomonas europaea*. This result is in agreement with the concept that microbial communities with a complex composition are commonly more robust than pure cultures. This is linked to the division of labour among species, which allows metabolic conversions impossible or difficult for a single strain, and the inter-species exchange of nutrients and metabolites involved in growth and cell-cell communication^14^.

The reactor community data also did not confirm hypothesis 1. F samples for Nitr-ur and OLAND, sharing the same *Nitrosomonas-*related OTU, showed no significant differences in ammonia oxidation compared to G23 samples (p > 0.1), while F samples for Nitr-au, containing AOA, even yielded an increased activity (p < 0.01) (Fig. 3d). Obtained biomass-specific nitritation rates are similar to those previously reported for these cultures^15,16^.

OLAND volumetric rates acted in line with hypothesis 2, showing a positive impact of refrigeration (p < 0.001). Yet, no positive refrigeration effect was observed for Nitr-au and Nitr-ur. Biomass specific rates also confute this hypothesis. For Nitr-ur, the lower microbial diversity (compared to OLAND) potentially contributed to this, in line with hypothesis 3.

#### Nitratation

For *Nitrobacter winogradskyi* (Fig. 3e), LEO exposure did not lower nitrite oxidation rates (p > 0.1), against hypothesis 1, and the presence of *Nitrosomonas europaea* and *Cupriavidus pinatubonensis* (CNsNb) even increased its rate (p < 0.01), despite having 1.6 times less cells (based on sample preparation), in the philosophy of hypothesis 3.

The reactor communities showed less clear trends comparing F to G23 samples. In case of *N. winogradskyi* as only or most dominant NOB, hypothesis 1 could neither be confirmed nor rejected. F samples were 1.2 times slower for OLAND (p < 0.01), despite of a relatively high diversity, or about 4 times faster for Nitr-ur (p < 0.001). The former is most in line with the observation of defined community CNsNb. In case of *Nitrospira sp*. as dominant NOB (Nitr-au), LEO exposure had a small positive impact (p < 0.1), opposing hypothesis 1.

When comparing G4 to G23 samples, the behaviour within the *N. winogradskyi* communities was similar to that between F and G23, rendering also inconclusive results for hypothesis 2. Indeed, G4 samples were 1.3 times slower for OLAND (p < 0.1), but about 600% faster for Nitr-ur (p < 0.001). With *Nitrospira sp*. as dominant NOB (Nitr-au), refrigeration yielded almost 400% activity of the reference G23 storage(p < 0.001), in line with hypothesis 2. More details are presented in Fig. 3f.

NOB are typically more susceptible to absolute high or low temperatures than AOB^17^. Hence, the high variability up to over 600%, as seen in the temperature profiles for F and G23 storage, could have caused the nitratation activity decline for Nitr-ur and Nitr-au. The higher microbial diversity in the OLAND sample may have exerted a stabilizing NOB effect (hypothesis 3).

#### Denitrification

Upon return to Earth, nitrate concentrations were considerably below the 500 mg NO_3_^-^N L^−1^ originally added to the storage medium. Since semi-quantitative test strip measurements required for the pre-incubation, revealed no indication of nitrite accumulation, nitrate depletion was interpreted as in-storage (and in-flight) denitrification activity. This being fully in line with the observations made in previous work on the OLAND culture^7^, in which a maximum concentration of 9.8 mg NO_2_-N L^−1^ was observed under similar terrestrial conditions, while using NO_3_^−^ as redox stabilizer. G23 samples presented the lowest residual nitrate levels (0–50 mg N L^−1^) and G4 the highest (89–113 mg N L^−1^) (Table S4.2), similar to previous findings^7^. This is likely linked to a lower availability of organic carbon for denitrification at 4 °C, due to a lower rate of microbial decay, as pointed out by the higher end-point biomass concentrations in G4 (S2).

To verify the internal availability of organic carbon during the storage period and the resultant activity of denitrifiers, endogenous denitrification assays were performed, as explained in detail in S1.5. Indeed, dosing solely nitrate to the biomass showed nitrate reducing activity for all cultures, with G23 samples mostly displaying the highest biomass-specific rates (Fig. S4.1).

The reactivation potential of denitrification was also investigated with sufficient methanol as external electron donor and sufficient carbon source to complete full denitrification, although intermediates and end products measurements were not performed. As methanol is typically used in conventional wastewater treatment, this could thus indicate the feasibility of using conventional biological processes during Space travel. Figure 3g shows the volumetric and relative denitrification reactivation potential measured as nitrate reducing activity. In contrast with hypothesis 1, the difference in volumetric and specific reactivation potential between G23 and F was not significant (p > 0.1). When comparing G23 to G4, Nitr-ur and Nitr-au showed specific nitrate reduction rates that were 3 times lower after refrigerated storage (Fig. S4.1). This is in line with abovementioned reasoning that higher temperatures unintendedly stimulated decay, and as such in-storage full denitrification activity and likely growth of denitrifiers. The experimental variability nonetheless did not lead to a statistical difference (p > 0.1), disproving hypothesis 2.

Overall, the results point towards the usefulness of nitrate as redox stabilizer suitable for Space application, as the losses of nitrogen gas are negligible compared to the nitrogen flows in RLSS. In a scenario that foresees in the treatment of the nitrogen secretion from a crew of 6 people (assuming 12 g-N person^−1^ d^−1^, 80% of which is excreted as urea)^18^ at typical urine nitrification conditions (loading rate 0.5 g N L^−1^ d^−1^,; biomass concentration 4 g VSS L^−1^ and protein/VSS conversion coefficient of 0.5) for the duration of the storage experiment (104 d); the rates observed indicates that the potential nitrogen loss due to full denitrification in the refrigerated samples will be less than 0.5%.

#### Anammox

OLAND was the only culture selected for its capacity of performing anammox. Hypothesis 1 and 2 were not confirmed, as F showed a similar rate than G23 and G4 conditions (Fig. 3h). The exposure to LEO conditions seems to have a lower impact than temperature, since the observed residual activity of 65 and 75% after a starvation period of 120 days at 20 and 23 °C are supported by the results for energy-deprived anammox^19,20^ or sulfide-related inhibition^21^. However, biomass-specific activity in G4 samples was similar to previously reported rates, when taking the community composition into account, indicating starvation as main cause of the reduced activity^14,19^.

## Conclusions

Even though the radiation was harsher in LEO than in ISS, hypothesis 1 (F < G23) was generally false, with only OLAND, holding *N. winogradskyi* as dominant NOB, displaying a slight (17%) decrease in nitratation activity. The general finding is even more striking, as many conversions even yielded higher activities after storage in Space (F > G23).

Hypothesis 2 predicted the best activity after refrigerated storage (G4 > G23), which was mostly true. The samples in which the highest activity was not observed after G4 conditions were Nitr-ur nitritation and OLAND nitritation and nitratation. Interestingly, the hypothesis was rejected for denitrification rates, yet confirmed for anammox.

For hypothesis 3, predicting a protective impact of an increased biodiversity, trends could not be generalized, even though interesting observations were made in the defined communities, where the presence of other bacteria increased nitritation and nitratation rates.

The presented data for the first time demonstrate the potential of five key conversions with their related sets of core microbes to contribute to essential nitrogen conversions for resource recovery in human Space exploration. Furthermore, from the results, a new working hypothesis can be recommended: refrigerated exposure to Space conditions can maximize the reactivation potential biological nitrogen cycle processes.

## Materials and Methods

### Preparation of the cultures

Eight cultures were selected (Table 1). Details on the cultivation and the entire materials and methods are given in Supporting Information S1.1. Active cultures were allowed to deplete the substrate in their respective medium before being aliquoted, in triplicates, into 5 mL Space-suitable cryotubes (VWR International, Radnor, USA). Preflight activities and protein concentration are presented in S2.

### Preservation conditions in Space and on Earth

The reference cultures preserved under refrigerated terrestrial conditions (G4) were kept in Ghent (BE) in cryotubes in a closed container. Other cultures were transported to Mol (BE), where the terrestrial cultures for storage at ambient temperature (G23) remained in a similar closed container.

The cultures intended for LEO exposure were handled in an identical manner to the reference ground cultures to ensure the imposed travel conditions would create the only difference. Each cryotube intended for LEO exposure (F) was equipped with temperature (iButton) and radiation (MTS-7) sensors^22^, and placed inside a hard plastic box. Figure 2 and Supporting Information S1.4 provides an overview of the flight conditions. The samples were transported to Moscow (Russia) on June 18^th^ 2014, and transferred to Baikonur (Kazakhstan). Samples were placed on separate locations in the capsule 1 day before the launch. The FOTON-M4 flight took off on July 18^th^, and landed on Earth after 44 days. The samples travelled back to Belgium, and reached Ghent by September 30^th^.

### Reactivation and activity tests

All activity tests were performed for each type of activity in at least triplicate, but in most cases in sixplicate in order to compensate for the miniaturized activity test and provide sufficient data for the required statistical analysis for hypothesis testing. Details on the reactivation procedure and activity tests are provided in the Supporting Information S1.5.

## Electronic supplementary material

Supporting Information
