# Supplementary material for: Nitrogen cycle microorganisms can be reactivated after Space exposure
Source: Sci Rep. 2018 Sep 13;8:13783. doi: 10.1038/s41598-018-32055-4 (PMC6137101; doi:10.1038/s41598-018-32055-4)
Supplement: Supplementary file 1 — Supporting Information [file 41598_2018_32055_MOESM1_ESM.pdf]

# Nitrogen cycle microorganisms can be reactivated after Space exposure

**Ralph E.F. Lindeboom[1,2,#], Chiara Ilgrande[1,#], José M. Carvajal-Arroyo[1], Ilse Coninx[3], Olivier Van Hoey[4], Hugo Roume[1,5], Julia Morozova[6], Kai M. Udert[7], Benedikt Sas[8], Christel Paille[9], Christophe Lasseur[9], Vyacheslav Ilyin[6], Peter Clauwaert[1], Natalie Leys[3,##] & Siegfried E. Vlaeminck[1,10,*,##]**

[1]Center for Microbial Ecology and Technology (CMET), Ghent University, Coupure Links 653, 9000 Gent, Belgium
[2]Section Sanitary Engineering, Department of Water Management, Faculty of Civil Engineering and Geosciences, Delft University of Technology, Stevinweg 1, 2628CN, Delft, The Netherlands
[3]Unit of Microbiology, Belgian Nuclear Research Centre (SCK•CEN), Boeretang 200, 2400 Mol, Belgium
[4]Unit of Research in Dosimetric Applications, Belgian Nuclear Research Centre (SCK•CEN), Boeretang 200, 2400 Mol, Belgium
[5] MetaGenoPolis, INRA, Université Paris-Saclay Domaine de Vilvert, Bat.325 78352, Jouy-en-Josas, France
[6]Institute of Biomedical Problems (IMBP), State Research Center of The Russian Federation, Khoroshevskoye Shosse, 76a, 123007 Moscow, Russia
[7]Eawag, Swiss Federal Institute of Aquatic Science and Technology, Überlandstrasse 133, 8600 Dübendorf, Switzerland
[8]Laboratory of Food Microbiology and Food Preservation, Ghent University, Coupure links 653, 9000 Gent, Belgium
[9]ESA/ESTEC Keplerlaan 1, 2201 AZ Noordwijk, The Netherlands
[10]Research of Sustainable Energy, Air and Water Technology, Department of Bioscience Engineering, University of Antwerp, Groenenborgerlaan 171, 2020 Antwerpen, Belgium

# Supplementary Information

* Corresponding author: siegfried.vlaeminck@uantwerpen.be
[#] equally contributed as first authors
[##] equally contributed as senior authors

# S1: Materials and Methods

**S1.1 Culture preparation**

C*upriavidus* 1245 was obtained from the Deutsche Sammlung von Mikroorganismen und Zellkulturen (DSM19553) and propagated in 284gluc medium in Erlenmeyers flasks at 30°C on a rotatory shaker at 120 rpm in the dark. *Nitrosomonas europaea* ATCC 19718 and *Nitrobacter winogradskyi* ATCC 25391 were obtained from SCK CEN. *Nitrosomonas europaea* ATCC 19718 was grown in ATCC medium 2265, while *Nitrobacter winogradskyi* ATCC 25391 in autotrophic medium (DSMZ medium 756c). *Nitrosomonas europaea* and *Nitrobacter winogradskyi* were allowed to consume all the substrate available, before sample preparation.

The axenic communities of C*upriavidus pinatubonensis* (C), *Nitrosomonas europaea* (Ns) and *Nitrobacter winogradskyi* (Nb) had an initial optical density ($OD_{600}$) of 1.07, 0.037 and 0.294, respectively. *Cupriavidus pinatubonensis* (C) cells were stored in C-free Tris-buffered mineral growth medium (MM284gluc medium), *Nitrosomonas europaea* (Ns) in ammonium free growth medium (ATCC 2265 Nitrosomonas europaea medium), and *Nitrobacter winogradskyi* (Nb) nitrite free growth medium (DMSZ 756c AUTOTROPHIC NITROBACTER medium).

The defined community NsNb was assembled mixing Ns and Nb cultures by mixing equal volumes of Ns culture at $OD_{600}$:0.022 and Nb culture at $OD_{600}$:0.118). The NsNbC community was assembled mixing equal volumes of Ns, Nb and C with a 1.0 : 6.3 : 25.3 $OD_{600}$-based ratio.

The reactor microbial community Nitr-ur (a community from a nitrifying Moving Bed Bio Reactor fed with urine)[1] was harvested from its carrier material (Kaldness K1) and placed inside the cryotubes in a concentration of 10.8 ± 0.07 g $L^{-1}$ Total Suspended Solid (TSS). The OLAND community  was harvested from a lab-scale Rotating Biological Contactor (RBC)[2] daily fed with $(NH_4)_2SO_4$ and placed in cryotubes at an average concentration 14.4 ± 0.04 g TSS $L^{-1}$. The Nitr-au community was collected from an aquaculture system fed with autotrophic  mineral medium containing urea and nitrite. It was diluted

twice due to the high TSS concentration in the original sample, to obtain a final concentration of 15 ± 0.03 g $L^{-1}$ [3,4]. 500 mg $NO_3^-$-N $L^{-1}$ was present in all storage media to stabilize redox-potential and prevent formation of sulphide by sulphate reduction[5]. Urea, $NH_4^+$ or $NO_2^-$ were fully depleted.

### S1.2 Contamination of axenic cultures and defined communities

Heterotrophic contamination was evaluated via colony count on LB agar plates. In the flight samples NsNb it was observed a contamination of $10^3$-$10^6$ cells/ml. These contaminants were identified as *Cupriavidus pinatubonensis*, *Acinetobacter* sp., *Frigoribacterium faeni* and *Staphylococcus* sp. The contamination was only observed in the flight samples, not in the ground samples. Although the exact cause of the contamination is not clear, it is possibly due to over- and underpressure during flight launch and return, causing some air and liquid to leave or enter the tubes, contaminating the samples. Contaminated samples were discarded from the activity test.

### S1.3 Molecular characterization of reactor communities

*DNA Extraction* was performed as described in the work of Coppens, et al. [6].

*Bacterial Illumina/16S rRNA gene sequencing.* The V3–V4 region of the bacterial 16S rRNA gene was sequenced with Illumina sequencing Miseq v3 Reagent kit (http://www.illumina.com/products/miseq-reagent-kit-v3.ilmn, by LGC Genomics GmbH, Berlin, Germany) using 2 x 300 bp paired-end reads and primers 341F-785R described in Stewardson et al.[7]

The PCR mixes included about 5 ng of DNA extract, 15 pmol of each forward primer 341F 5’-NNNNNNNNTCCTACGGGNGGCWGCAG and reverse primer 785R 5’- NNNNNNNNTGACTACH VGGGTATCTAAKCC in 20 µL volume of MyTaq buffer containing 1.5 units MyTaq DNA polymerase (Bioline) and 2 µl of BioStabII PCR Enhancer. [8 8 8 16] For each sample, the forward and reverse primers had the same 8-nt barcode sequence. PCRs were carried out for 30 cycles using the following parameters: 2 min 96 °C pre-denaturation; 96 °C for 15 s, 50 °C for 30 s, 72 °C for 60 s. DNA concentration of amplicons of interest was determined by gel electrophoresis. About 20 ng amplicon

DNA of each sample were pooled for up to 48 samples carrying different barcodes. PCRs showing low yields were further amplified for 5 cycles. The amplicon pools were purified with one volume AMPure XP beads (Agencourt) to remove primer dimer and other small mispriming products, followed by an additional purification on MinElute columns (Qiagen). About 100 ng of each purified amplicon pool DNA was used to construct Illumina libraries using the Ovation Rapid DR Multiplex System 1-96 (NuGEN). Illumina libraries were pooled and size selected by preparative gel electrophoresis. Sequencing was done on an Illumina MiSeq using v3 Chemistry (Illumina).

Bioinformatics were conducted with metagenomics analysis (QIIME).[9] Data were pre-processing by first demultiplexing of all samples using Illumina's CASAVA data analysis software v1.8.2 The reads were then sorted by amplicon inline barcodes, no barcode mismatches were allowed, the barcode sequence was clipped from the sequence after sorting and reads with missing barcodes, one-sided barcodes or conflicting barcode pairs were discarded. Sequencing adapters in all reads were removed and read with final length below 100 nt were discarded (Clipping of Illumina TruSeq$^{TM}$ adapters in all reads). Combination of forward and reverse reads was done using BBMerge 34.30 (http://bbmap.sourceforge.net/). The sequence fragments were turned into forward-reverse primer orientation after removing the primer sequences. FastQC report was creating for every FASTQ files. 16S pre-processing and OTU picking from amplicons were performed using Mothur v1.35.1 software package [10]. Sequences containing ambiguous bases (Ns), with homo-polymer stretches of more than 8 bases or with an average Phred quality score below 33 were removed. Read were align against the 16S Silva reference alignment v119 Truncated and unspecific PCR products were removed. 15 000 sequences per sample were subsampling. Pre-clustering allow up to 3 differing based in a cluster. Chimera were removed using uchime algorithm[11]. Taxonomical classification of sequences and removal of non-bacterial sequences were done using Silva database. OTU were picking by clustering at the 97 % identity level using the cluster split method.

*Archaeal Illumina/16S rRNA gene sequencing.* The archaea 16S rRNA gene was sequenced with Illumina sequencing Miseq v3 Reagent kit (http://www.illumina.com/products/miseq-reagent-kit-

v3.ilmn, by LGC Genomics GmbH, Berlin, Germany) using 2 x 300 bp paired-end reads and primers A340F [12] and A915R described in Delong. [13]

## S1.4 Dosimetric calculations and flight characteristics

The received daily radiation, based on the MTS-7 (LEO-exposure) and MCP-6 (background radiation) , was calculated as previously described.[14] An overview of the FOTON-M4 flight conditions are presented in table S1.1.

**Table S1.1: FOTON-M4 flight characteristics**

| | |
|---|---|
| Flight duration | 44 days (July 18th 2014 - September 1st 2014) |
| Altitude | 258-571 km; 64.9◦ inclination towards Earth surface |
| Gravity (g) | $10^{-3}$-$10^{-4}$ (estimated [15]) |

## S1.5 Reactivation and activity tests

Upon retrieval and prior to reactivation the culture media was evaluated for residual concentrations of nitrate, nitrite and ammonium using Semi-quantitative test strips (Macherey Nagel, Germany). Reactivation of all the microbial communities was then performed by adding a substrate medium consisting of 50 mg $NO_2^-$-N or $NH_4^+$-N $L^{-1}$ buffered at a pH of 7 with 0.011 g $L^{-1}$ $KH_2PO_4$, 0.014 g $L^{-1}$ $K_2HPO_4$ and 0.0025 g $L^{-1}$ $NaHCO_3$. The activity tests on the natural microbial communities could be performed as soon as the substrate was depleted, 3 days after the dosing the initial substrate medium. The axenic and the defined microbial communities required a re-activation of 10 days to completely consume the substrate, due to slower substrate conversion rates. The reactivation resulted in a 2 times dilution of the different cultures.

Activity tests were performed evaluating the conversion of the different nitrogen species according to the methods reported in S1.6. The different metabolic conversions were tested separately. Ureolysis was evaluated as ammonia production in presence of 250 mg-N $L^{-1}$ of allylthiourea, an inhibitor of ammonia oxidation. Nitritation and anammox rates were calculated monitoring ammonia concentration over time, while nitratation rates were calculated by monitoring nitrite concentration. The nitrate reducing activity, used as an indicator for denitrification, was evaluated by measuring

nitrate concentrations over time. Denitrification Intermediates $NO_2^-$, $NO_2$, NO, $N_2O$ and the final product $N_2$ and $NH_4^+$ were not quantified.

To perform the activity tests, the reactivated biomass was additionally diluted 2.5 times in the same medium used for the reactivation containing 50 mg-N $L^{-1}$ of substrate. This resulted in a total 5-fold final dilution.

Ureolysis, nitritation and nitratation were tested in in flat-bottom 96-well plates. For each cryotube, 250µL duplicates were prepared, resulting in a sixplicate for each culture. The plates were then placed in a plate incubator (MB100-4A, Thermoshakers Aosheng, China) at 28°C at 600 rpm for up to 6 days or until complete consumption of the substrate. Denitrification and anammox activity tests were performed in triplicates in 10 mL penicillin bottles incubated at 28°C and shaken at 120 rpm. For the denitrification assay with external carbon, methanol was dosed at 1.91 g methanol $g^{-1}$ $NO_3^-$-N (COD/N = 3.8).

The activity of each culture was normalized towards the reference scenario, the G23 ground storage of that same culture and plotted on the secondary y-axis of figure 3a-f to enable the visual comparison between the different assays of all the different communities.

**S1.6 Analytical methods**

*Protein* concentration of all reactor microbial communities was determined using the Lowry method.[16] Bovine serum albumin (BSA) standards were used for calibration.

*Ammonium, nitrite and nitrate* concentration were evaluated spectrophotometrically. The ammonium concentration was determined by the Berthelot reaction[17], the nitrite concentration was determined by the Montgomerey reaction[18] while the nitrate concentration was determined as described by Cataldo et al.[19]. All spectrophotometric measurements were performed using a Microplate Readers Infinite® F50 (Tecan group Ltd., Germany).

Both R and MSExcel were used for dataprocessing. After all concentrations were measured, values were uploaded automatically in MSExcel by the plate reader. From here they were processed into a format suitable for R version 3.4.0 (The R Foundation for Statistical Computing). The package plyr was used to summarize evaluate the data, whereas ggplot2 was used for preparing the activity plots and linear curve fitting of the volumetric rates. Data were grouped into linear models and compared using ANOVA. Normal distribution of residuals were evaluated visually using a QQ-plot and Shapiro test.

## References


1 Etter, B., Hug, A. & Udert, K. M. Total nutrient recovery from urine–operation of a pilot-scale nitrification reactor. *WEF/IWA International Conference on Nutrient Removal and Recovery, 2013: Trends in Resource Recovery and Use* 28-31 (2013).

2 Kerckhof, F.-M. *et al.* Optimized cryopreservation of mixed microbial communities for conserved functionality and diversity. *PloS one* **9.6** (2014).

3 Rombaut, G. *et al.* Improved performance of an intensive rotifer culture system by using a nitrifying inoculum (ABIL). *Aquaculture Research* **34**, 165-174 (2003).

4 Grommen, R., Van Hauteghem, I., Van Wambeke, M. & Verstraete, W. An improved nitrifying enrichment to remove ammonium and nitrite from freshwater aquaria systems. *Aquaculture* **211**, 115-124 (2002).

5 Vlaeminck, S., Geets, J., Vervaeren, H., Boon, N. & Verstraete, W. Reactivation of aerobic and anaerobic ammonium oxidizers in OLAND biomass after long-term storage. *Appl Microbiol Biotechnol* **74**, 1376-1384 (2007).

6 Coppens, J. *et al.* Nitrification and microalgae cultivation for two-stage biological nutrient valorization from source separated urine. *Bioresource Technology* **211**, 41-50 (2016).

7 Stewardson, A. J., Harbarth, S. & Graves, N. Valuation of hospital bed-days released by infection control programs: a comparison of methods. *Infection Control & Hospital Epidemiology* **35**, 1294-1297 (2014).

8 Prokopenko, M. G. *et al.* Nitrogen losses in anoxic marine sediments driven by Thioploca-anammox bacterial consortia. *Nature* **500**, 194-198 (2013).

9 Caporaso, J. G. *et al.* QIIME allows analysis of high-throughput community sequencing data. *Nature methods* **7**, 335-336 (2010).

10 Schloss, P. D. *et al.* Introducing mothur: open-source, platform-independent, community-supported software for describing and comparing microbial communities. *Applied and environmental microbiology* **75**, 7537-7541 (2009).

11 Edgar, R. C., Haas, B. J., Clemente, J. C., Quince, C. & Knight, R. UCHIME improves sensitivity and speed of chimera detection. *Bioinformatics* **27**, 2194-2200 (2011).

12 Gantner, S., Andersson, A. F., Alonso-Sáez, L. & Bertilsson, S. Novel primers for 16S rRNA-based archaeal community analyses in environmental samples. *Journal of Microbiological Methods* **84**, 12-18 (2011).

13 DeLong, E. F. Archaea in coastal marine environments. *Proceedings of the National Academy of Sciences* **89**, 5685-5689 (1992).

14 Goossens, O. *et al.* Radiation dosimetry for microbial experiments in the International Space Station using different etched track and luminescent detectors. *Radiation protection dosimetry* **120**, 433-437 (2006).

15 Horneck, G., Klaus, D. M. & Mancinelli, R. L. Space microbiology. *Microbiology and Molecular Biology Reviews* **74**, 121-156 (2010).

16 Lowry, O. H., Rosebrough, N. J., Farr, A. L. & Randall, R. J. Protein measurement with the Folin phenol reagent. *J biol Chem* **193**, 265-275 (1951).

17 Bucur, B., Catala Icardo, M. & Martinez Calatayud, J. Spectrophotometric determination of ammonium by an rFIA assembly. *Revue Roumaine de Chimie* **51**, 101 (2006).

18 Montgomery, H. C., andJ. F. Dymock. 1961. The determination of nitrite in water. *Analyst (London)* **86**, 414-416.

19 Cataldo, D., Maroon, M., Schrader, L. & Youngs, V. Rapid colorimetric determination of nitrate in plant tissue by nitration of salicylic acid 1. *Communications in Soil Science & Plant Analysis* **6**, 71-80 (1975).

# S2: Preflight activity and biomass concentrations

**Axenic and defined communities'** metabolic activity was tested upon samples submission (one month before the actual LEO-flight), to confirm strain activity. *Cupriavidus pinatubonensis* was responsible for the ureolytic activity in CNsNb culture of 3.1 ± 0.7 mg urea-N $L^{-1}$ $d^{-1}$. Nitritation rates were 1.7 ± 0.9, 0.4 ± 0.7 and 8.8 ± 4.3 mg $NH_4^+$-N $L^{-1}$ $d^{-1}$ for Ns, NsNb and CNsNb respectively. Nitratation rates were 92.6 ± 65.6, 190.7 ± 9.0 and 93.0 ± 78 mg $NO_2^-$-N $L^{-1}$ $d^{-1}$ for Nb, NsNb and CNsNb respectively.

**Reactor microbial communities** exhibited all expected metabolic activities. The pre-flight ureolytic rates were 5.4, 10.9 and 129.6 mg $NH_4^+$-N $L^{-1}$ $d^{-1}$ for Nitr–ur, OLAND and Nitr–au. Nitritation rates were 17.1, 33.0 and 16.3 mg $NH_4^+$-N $L^{-1}$ $d^{-1}$, and nitratation rates were 27.4, 30.9 and 35.0 mg $NO_2^-$-N $L^{-1}$ $d^{-1}$. Interestingly, Nitr–ur, that originated from a urine fed reactor, presented the lowest ureolytic activity.

**Protein concentration** was evaluated after ca. 3 months storage (at 4°C and at 23°C on Earth, at LEO) for the reactor communities. Axenic and defined culture protein levels were below detection limit. Among them, OLAND presented the highest protein content while Nitr-au the lowest. F and G23 samples showed a decrease in protein concentrations compared to the 4°C, likely the result of a faster decay under higher storage temperatures. The higher biomass content of the reactor microbial communities is most likely responsible of higher volumetric rates (Figure 3) and shorter reactivation time (S1.5) compared to the axenic and defined communities.

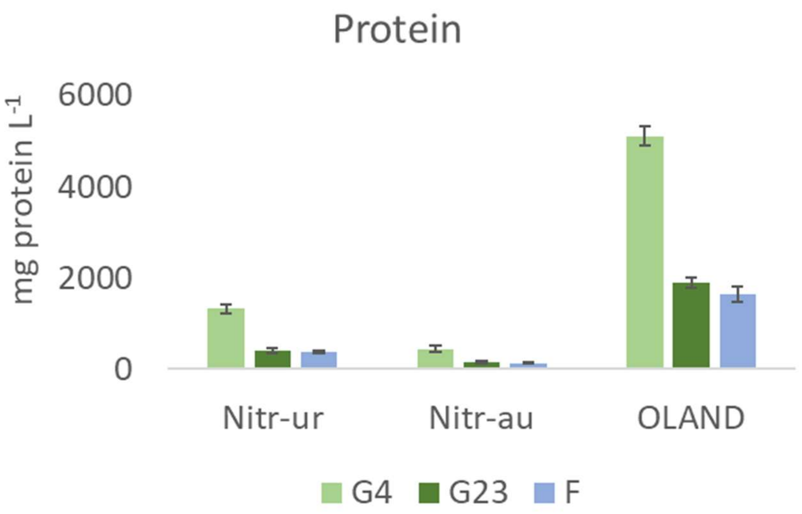


**Figure S2.1: Overview of the protein concentrations of the reactor communities' samples during the preservation on ground at 4°C (G4), 23°C (G23), at in low Earth orbit (LEO) flight (F).**

# S3: Microbial community composition

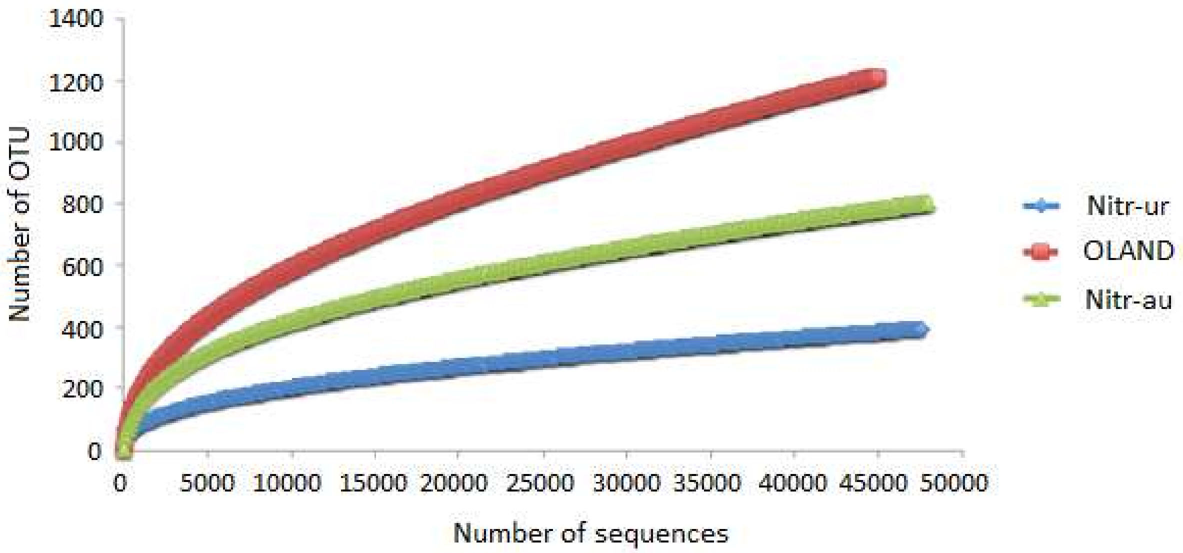


**Figure S3.1: Rarefaction curves of the sequencing dataset**

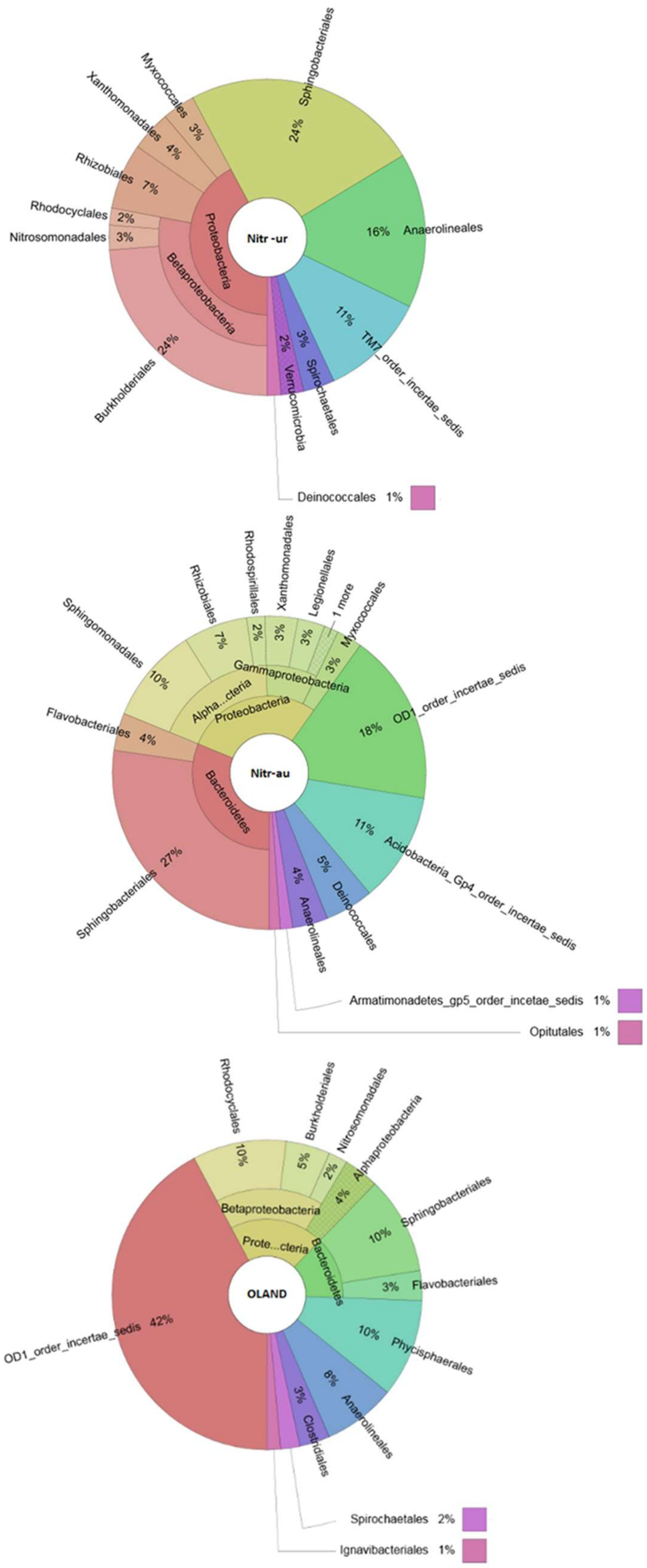


**Figure S3.2 Composition of the reactor communities in Nitr-ur, OLAND and Nitr-au samples after exposure to LEO conditions (abundance of populations representing more than 1% of the community).**

**Table S3.1: Overview of OTU retrieved from the samples after LEO-exposure.**

| #OTU ID | OTU count number | | | Consensus lineage (Mothur) | Closest neighbour | Query cover | E-value | Identity | Accession |
|---|---|---|---|---|---|---|---|---|---|
| | **Nitr-ur** | **Nitr-au** | **OLAND** | | **NCBI - Blastn** | | | | |
| | | | | | | | | | |
| Otu0028 | 1039 | 0 | 346 | *Nitrosomonas sp.* | *Nitrosomonas europaea* | 100% | 2e-73 | 98% | KU860549.1 |
| Otu0001 (Archaea) | 0 | 80000 | 0 | *Thaumarcheota* | *Nitrososphaera* | 100% | 0.0 | 96% | JF748724.1 |
| | | | | | | | | | |
| Otu0014 | 347 | 682 | 2 | *Nitrobacter sp.* | *Nitrobacter winogradskyi strain* Nb-255 | 100% | 0.0 | 100% | NR_074324.1 |
| Otu0240 | 0 | 7 | 28 | *Nitrospira sp.* | *Nitrospira moscoviensis* M-1 | 100% | 2e-157 | 91% | CP011801.1 |
| | | | | | Candidatus *Nitrospira defluvii* | 100% | 3e-167 | 92% | NR_074700.1 |
| Otu1711 | 0 | 23 | 0 | *Nitrospira sp.* | Candidatus *Nitrospira defluvii* | 100% | 0.0 | 97% | NR_074700.1 |
| | | | | | | | | | |
| Otu0017 | 0 | 0 | 3,572 | Unclasssified Bacteria | Candidatus *Kuenenia stuttgartiensis (KUST)* | 99% | 0.0 | 82% | CT573071.1 |
| Otu0021 | 0 | 0 | 1,622 | Unclasssified Bacteria | Candidatus *Kuenenia stuttgartiensis* | 99% | 1e-92 | 82% | JQ889383.1 |
| | | | | | | | | | |
| Otu0001 | 9211 | 4 | 2 | *Comamonas sp.* | *Comamonas sp.* MPI12 | 100% | 0.0 | 99% | FN430655.1 |
| Otu0042 | 750 | 1 | 3 | *Hyphomicrobium sp.* | *Hyphomicrobium denitrificans strain* FJNU-6 | 100% | 0.0 | 99% | KJ541680.1 |

| | | | | | | | | | |
|---|---|---|---|---|---|---|---|---|---|
| Otu0057 | 0 | 118 | 156 | Unclassified Rhizobiales | *Rhodoplanes sp.* | 100% | 0.0 | 99% | HM769667.1 |
| Otu0126 | 0 | 133 | 8 | Unclassified Rhodospirillaceae | *Azospirillum sp.* J8 | 100% | 7e-161 | 92% | KU870754.1 |
| Otu0201 | 0 | | 146 | Unclassified Rhodospirillaceae | *Azospirillum melinis strain* BJ-1 | 100% | 7e-166 | 93% | KX022950.1 |
| Otu0334 | 0 | | 89 | *Pseudomonas* sp. | *Pseudomonas xinjiangensis* strain Y61 | 100% | 0.0 | 99% | KU601275.1 |
| Otu0536 | 0 | | 0 | *Pseudomonas* sp. | *Pseudomonas anguilliseptica* VITEPRRL6 | 100% | 0.0 | 100% | KR149276.1 |
| Otu0063 | 0 | | 558 | Unclassified Flavobacteriaceae | Muricauda sp. DG1233 | 100% | 7e-168 | 92% | DQ486480.1 |
| Otu0072 | 0 | | 1,636 | Unclassified Sphingobacteriales | *Lewinella cohaerens* | 100% | 5e-163 | 91% | KF228160.1 |

Phylogenetic trees were constructed to acquire more information on a species level for the OTUs related to nitrite oxidizing bacteria (NOB; Figure S4.2), ammonia oxidizing archaea (AOA; Figure S4.3), and anaerobic ammonium oxidizing bacteria (anammox; Figure S4.4) and putative denitrifying bacteria (Figure S4.5).

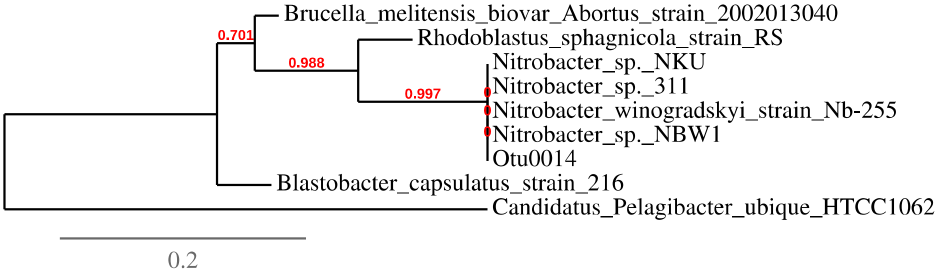


**Figure S3.3: Phylogenetic tree for OTU14 (NOB). Number of individual branch measure the dissimilarity between sequences and scale measure the number of substitution per site.**

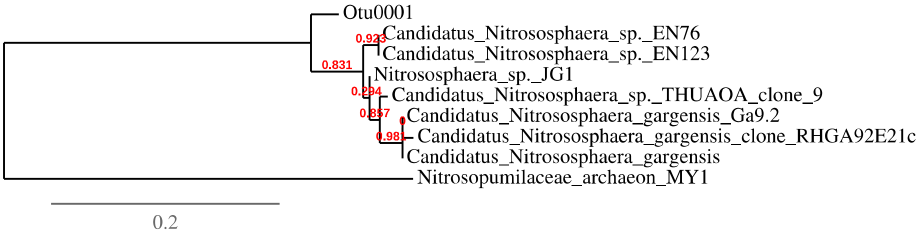


**Figure S3.4: Phylogenetic tree for OTU1 (AOA). Number of individual branch measure the dissimilarity between sequences and scale measure the number of substitution per site.**

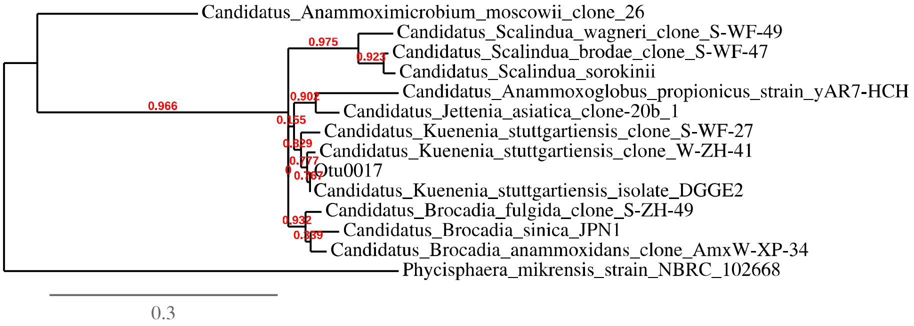


**Figure S3.5: Phylogenetic tree for OTU17 (anammox). Number of individual branch measure the dissimilarity between sequences and scale measure the number of substitution per site.**

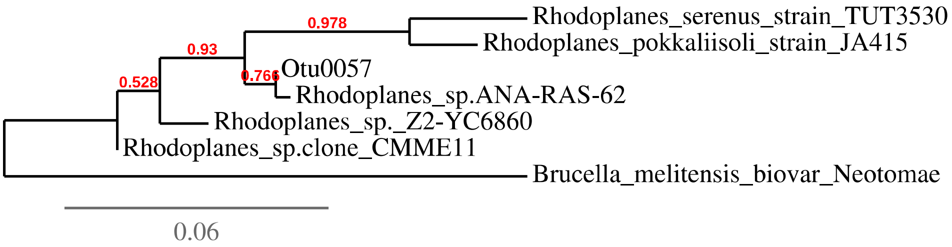


**Figure S3.6: Phylogenetic tree for OTU57 (putative denitrifier). Number of individual branch measure the dissimilarity between sequences and scale measure the number of substitution per site.**

# S4: Activity tests – Volumetric and biomass-specific rates

**Table S4.1: Comparison of volumetric and biomass specific activity after reactivation for cultures preserved on ground at 4°C (G4), on ground at 23°C (G23) and in low Earth orbit flight (F). Ureolysis was measured as ammonium production, nitritation as ammonium consumption, nitratation as nitrite consumption, denitrification as nitrate consumption and anammox activity as ammonium consumption. ND: activity not determined; NA: activity potentially present but not determined.**

| | | Volumetric rates mg N $L^{-1}$ $d^{-1}$ | | | | | | | | Biomass-specific rates mg N $g^{-1}$ protein $d^{-1}$ | | | | | |
|---|---|---|---|---|---|---|---|---|---|---|---|---|---|---|---|
| | | G4 | | G23 | | F | | | | G4 | | G23 | | F | |
| | | Avg | Stdev | Avg | Stdev | Avg | Stdev | F vs G23 | G4 vs G23 | Avg | Stdev | Avg | Stdev | Avg | Stdev |
| **Ureolysis** | **C** | NA | NA | 13 | 1 | 13 | 1 | p>0.1 | ND | NA | NA | NA | NA | NA | NA |
| | **Ns** | ND | ND | ND | ND | ND | ND | ND | ND | ND | ND | ND | ND | ND | ND |
| | **Nb** | ND | ND | ND | ND | ND | ND | ND | ND | ND | ND | ND | ND | ND | ND |
| | **NsNb** | ND | ND | ND | ND | ND | ND | ND | ND | ND | ND | ND | ND | ND | ND |
| | **CNsNb** | NA | NA | 13 | 2 | 12 | 2 | p>0.1 | ND | NA | NA | NA | NA | NA | NA |
| | **Nitr-ur** | 70 | 7 | 29 | 14 | 25 | 7 | p>0.1 | p<0.05 | 54 | 5 | 66 | 32 | 57 | 16 |
| | **Nitr-au** | 944 | 113 | 163 | 112 | 714 | 99 | p<0.001 | p>0.1 | 2187 | 262 | 1019 | 700 | 6492 | 900 |
| | **OLAND** | 180 | 15 | 83 | 9 | 88 | 8 | p>0.1 | p<0.001 | 221 | 18 | 45 | 5 | 54 | 5 |
| **Nitritation** | **C** | ND | ND | ND | ND | ND | ND | ND | ND | ND | ND | ND | ND | ND | ND |
| | **Ns** | NA | NA | 2 | 1 | 1 | 1 | p>0.1 | ND | NA | NA | NA | NA | NA | NA |
| | **Nb** | ND | ND | ND | ND | ND | ND | ND | ND | ND | ND | ND | ND | ND | ND |
| | **NsNb** | NA | NA | 3 | 1 | 7 | 2 | p>0.1 | ND | NA | NA | NA | NA | NA | NA |
| | **CNsNb** | NA | NA | 9 | 5 | 9 | 5 | p>0.1 | ND | NA | NA | NA | NA | NA | NA |
| | **Nitr-ur** | 103 | 7 | 140 | 13 | 138 | 21 | p>0.1 | p<0.05 | 80 | 5 | 184 | 17 | 184 | 28 |
| | **Nitr-au** | 188 | 14 | 90 | 34 | 153 | 12 | p<0.01 | p>0.1 | 433 | 32 | 851 | 322 | 958 | 75 |
| | **OLAND** | 531 | 30 | 264 | 10 | 248 | 7 | p>0.1 | p<0.001 | 103 | 6 | 180 | 7 | 171 | 5 |
| **Nitratation** | **C** | ND | ND | ND | ND | ND | ND | ND | ND | ND | ND | ND | ND | ND | ND |
| | **Ns** | ND | ND | ND | ND | ND | ND | ND | ND | ND | ND | ND | ND | ND | ND |
| | **Nb** | NA | NA | 102 | 10 | 97 | 8 | p>0.1 | ND | NA | NA | NA | NA | NA | NA |

| | | | | | | | | | | | | | | | |
|---|---|---|---|---|---|---|---|---|---|---|---|---|---|---|---|
| | **NsNb** | NA | NA | 95 | 13 | 89 | 4 | p>0.1 | ND | NA | NA | NA | NA | NA | NA |
| | **CNsNb** | NA | NA | 82 | 8 | 132 | 28 | p<0.01 | ND | NA | NA | NA | NA | NA | NA |
| | **Nitr-ur** | 243 | 12 | 39 | 17 | 145 | 5 | p<0.001 | p<0.001 | 186 | 9 | 66 | 29 | 210 | 7 |
| | **Nitr-au** | 512 | 8 | 136 | 45 | 150 | 4 | p<0.1 | p<0.001 | 1187 | 19 | 1232 | 408 | 1399 | 37 |
| | **OLAND** | 66 | 16 | 86 | 3 | 71 | 8 | p<0.01 | p<0.1 | 14 | 3 | 62 | 2 | 43 | 5 |
| **Denitrification** | **C** | NA | NA | NA | NA | NA | NA | ND | ND | NA | NA | NA | NA | NA | NA |
| | **Ns** | ND | ND | ND | ND | ND | ND | ND | ND | ND | ND | ND | ND | ND | ND |
| | **Nb** | ND | ND | ND | ND | ND | ND | ND | ND | ND | ND | ND | ND | ND | ND |
| | **NsNb** | ND | ND | ND | ND | ND | ND | ND | ND | ND | ND | ND | ND | ND | ND |
| | **CNsNb** | NA | NA | NA | NA | NA | NA | ND | ND | NA | NA | NA | NA | NA | NA |
| | **Nitr-ur** | 16 | 5 | 18 | 7 | 17 | 4 | p>0.1 | p>0.1 | 129 | 5 | 741 | 19 | 641 | 10 |
| | **Nitr-au** | 4 | 3 | 9 | 11 | 19 | 8 | p>0.1 | p>0.1 | 127 | 6 | 1216 | 99 | 2554 | 72 |
| | **OLAND** | 12 | 3 | 9 | 4 | 13 | 2 | p>0.1 | p>0.1 | 35 | 1 | 35 | 3 | 68 | 2 |
| **Anammox** | **C** | ND | ND | ND | ND | ND | ND | ND | ND | ND | ND | ND | ND | ND | ND |
| | **Ns** | ND | ND | ND | ND | ND | ND | ND | ND | ND | ND | ND | ND | ND | ND |
| | **Nb** | ND | ND | ND | ND | ND | ND | ND | ND | ND | ND | ND | ND | ND | ND |
| | **NsNb** | ND | ND | ND | ND | ND | ND | ND | ND | ND | ND | ND | ND | ND | ND |
| | **CNsNb** | ND | ND | ND | ND | ND | ND | ND | ND | ND | ND | ND | ND | ND | ND |
| | **Nitr-ur** | ND | ND | ND | ND | ND | ND | ND | ND | ND | ND | ND | ND | ND | ND |
| | **Nitr-au** | ND | ND | ND | ND | ND | ND | ND | ND | ND | ND | ND | ND | ND | ND |
| | **OLAND** | 27 | 14 | 6 | 4 | 10 | 4 | p>0.1 | p>0.1 | 93 | 10 | 21 | 3 | 31 | 2 |

**Table S4.2** **Residual nitrate concentration (mg $NO_3^-$-N $L^{-1}$) after G4, G23 and F storage for 104 days, derived consumption rates (mg $NO_3^-$-N $L^{-1}$ $d^{-1}$) and biomass specific consumption rates (mg $NO_3^-$-N g $protein^{-1}$ $d^{-1}$), based on the original supplementation of 500 mg $NO_3^-$-N $L^{-1}$ and the end-point biomass level (g protein $L^{-1}$, S2).**

| | **G4** | | | | | | **G23** | | | | | | **F** | | | | | |
|---|---|---|---|---|---|---|---|---|---|---|---|---|---|---|---|---|---|---|
| | **Residual $NO_3^-$** | | **Volumetric consumption rates** | | **Biomass-specific consumption rates** | | **Residual $NO_3^-$** | | **Volumetric consumption rates** | | **Biomass-specific consumption rates** | | **Residual $NO_3^-$** | | **Volumetric consumption rates** | | **Biomass-specific consumption rates** | |
| | Avg | Stdev | Avg | Stdev | Avg | Stdev | Avg | Stdev | Avg | Stdev | Avg | Stdev | Avg | Stdev | Avg | Stdev | Avg | Stdev |
| **Nitr-ur** | 105.4 | 21.0 | 3.8 | 0.2 | 2.9 | 0.2 | 0.0 | 0.0 | > 4.8 | - | > 12.0 | - | 70.3 | 25.5 | 4.4 | 0.2 | 11.2 | 0.7 |
| **Nitr-au** | 112.9 | 16.6 | 3.7 | 0.2 | 8.4 | 0.4 | 50.0 | 30.7 | 4.3 | 0.3 | 28.2 | 1.9 | 84.7 | 22.8 | 1.0 | 0.2 | 31.5 | 1.7 |
| **OLAND** | 89.2 | 32.1 | 4.0 | 0.3 | 0.8 | 0.1 | 1.6 | 1.0 | 4.8 | 0.01 | 2.5 | 0.01 | 67.1 | 10.0 | 4.2 | 0.1 | 2.5 | 0.1 |

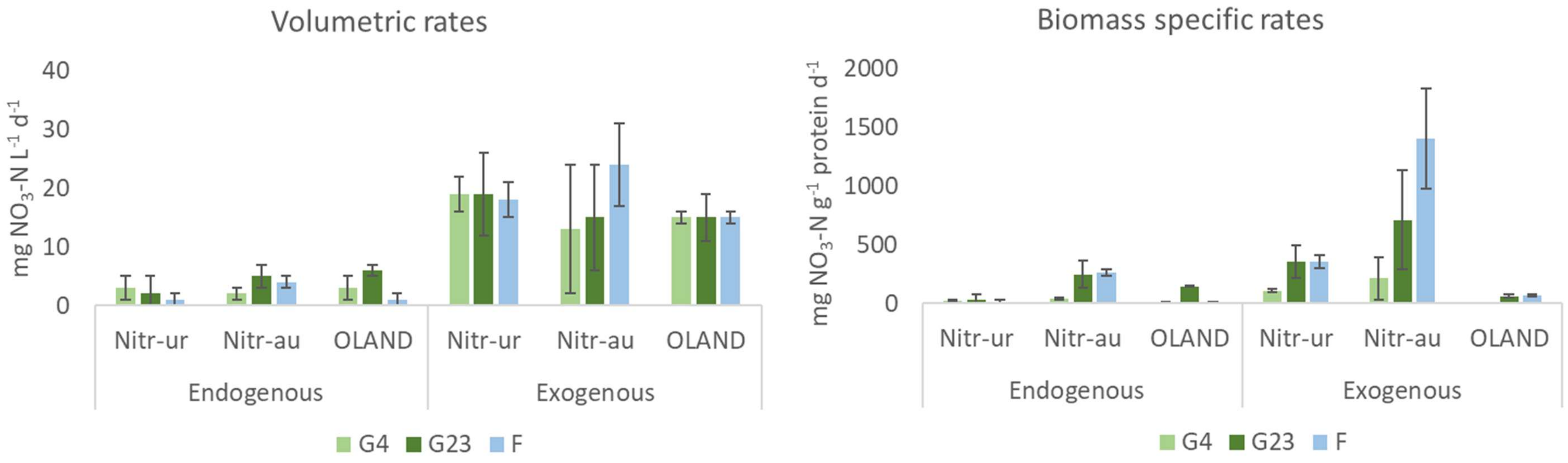


**Figure S4.1 Overview of the volumetric (a) and biomass specific (b) endogenous (biomass-driven) and exogenous (methanol-driven) denitrification activity after G4, G23 and F storage measured as nitrate consumption rates.**

# S5: Activity tests - Measured substrate concentrations

Overview of the ammonium and nitrite concentration over time of activity tests for axenic, defined and reactor cultures for ureolysis, nitritation, nitratation, anammox. Denitrification was measured by nitrate addition and depletion under conditions with only an internal carbon source (endogenous denitrification) or in presence of an additional external carbon source (3.8 g COD/g N).

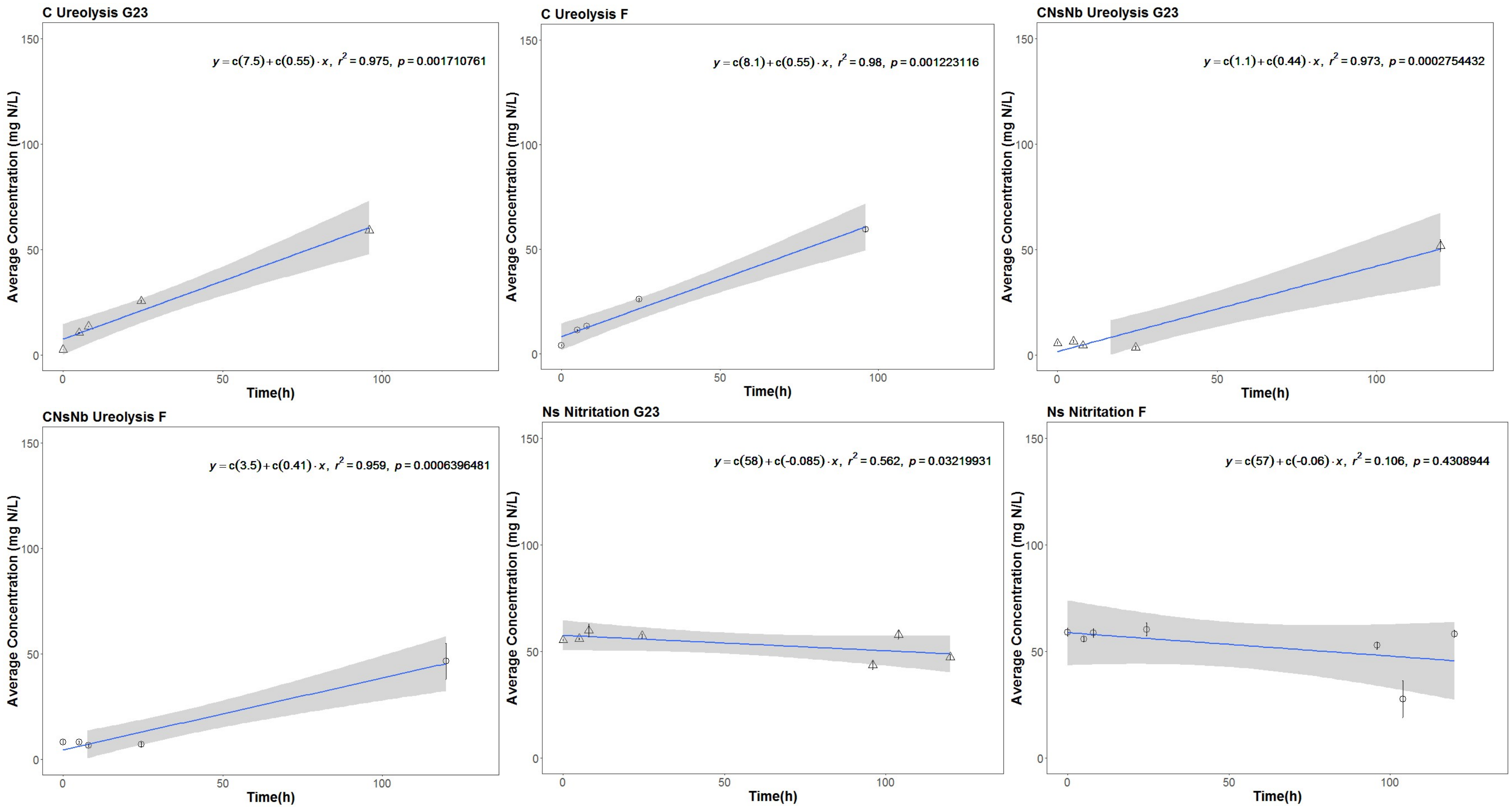

C Ureolysis G23
y = c(7.5) + c(0.55) · x, r² = 0.975, p = 0.001710761
C Ureolysis F
y = c(8.1) + c(0.55) · x, r² = 0.98, p = 0.001223116
CNsNb Ureolysis G23
y = c(1.1) + c(0.44) · x, r² = 0.973, p = 0.0002754432
CNsNb Ureolysis F
y = c(3.5) + c(0.41) · x, r² = 0.959, p = 0.0006396481
Ns Nitritation G23
y = c(58) + c(-0.085) · x, r² = 0.562, p = 0.03219931
Ns Nitritation F
y = c(57) + c(-0.06) · x, r² = 0.106, p = 0.4308944
Average Concentration (mg N/L)
Time(h)
0
50
100
150

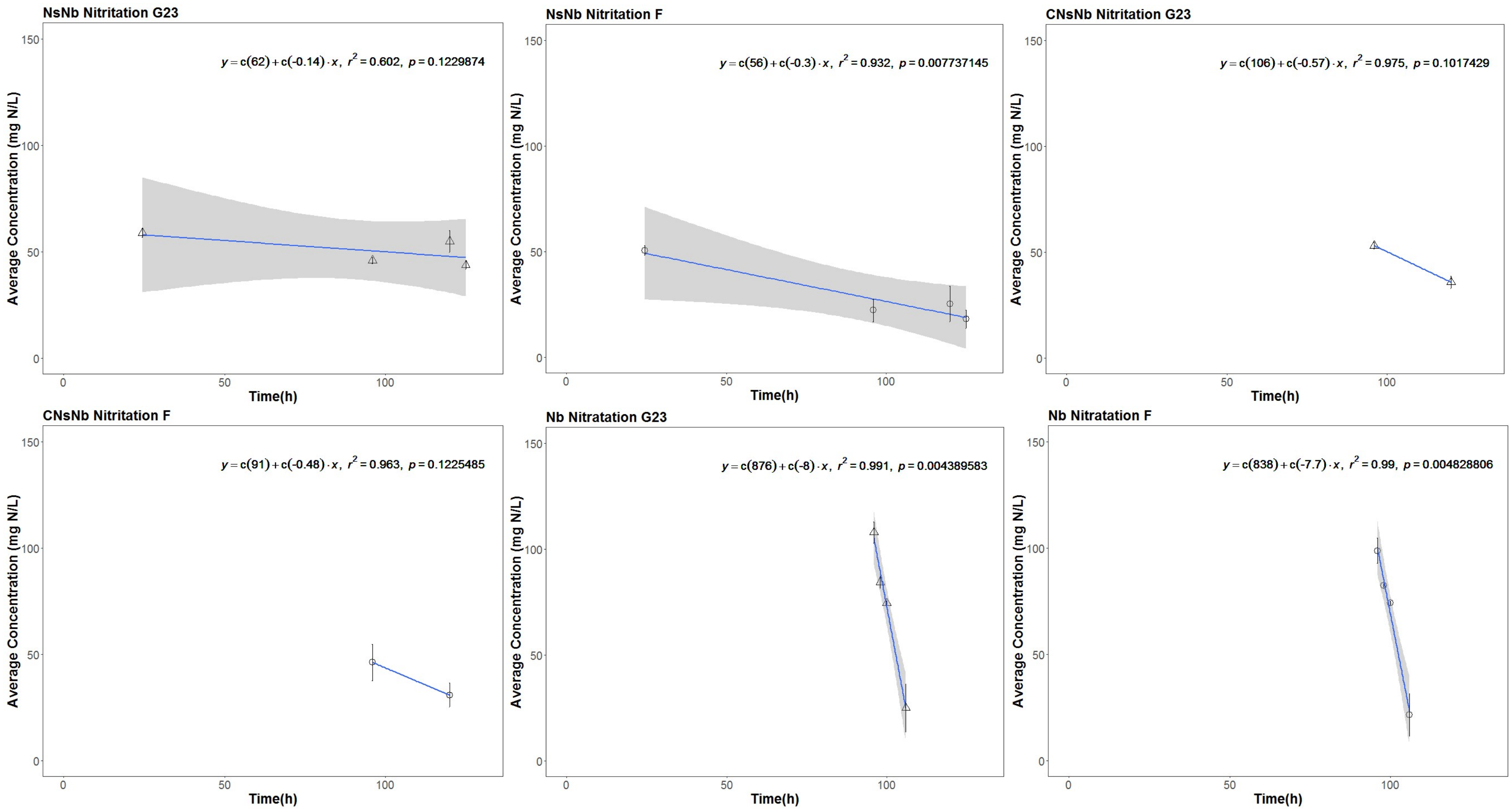

NsNb Nitritation G23
$y = c(62) + c(-0.14) \cdot x,\ r^2 = 0.602,\ p = 0.1229874$
NsNb Nitritation F
$y = c(56) + c(-0.3) \cdot x,\ r^2 = 0.932,\ p = 0.007737145$
CNsNb Nitritation G23
$y = c(106) + c(-0.57) \cdot x,\ r^2 = 0.975,\ p = 0.1017429$
CNsNb Nitritation F
$y = c(91) + c(-0.48) \cdot x,\ r^2 = 0.963,\ p = 0.1225485$
Nb Nitratation G23
$y = c(876) + c(-8) \cdot x,\ r^2 = 0.991,\ p = 0.004389583$
Nb Nitratation F
$y = c(838) + c(-7.7) \cdot x,\ r^2 = 0.99,\ p = 0.004828806$
Average Concentration (mg N/L)
Time(h)
0
50
100
150

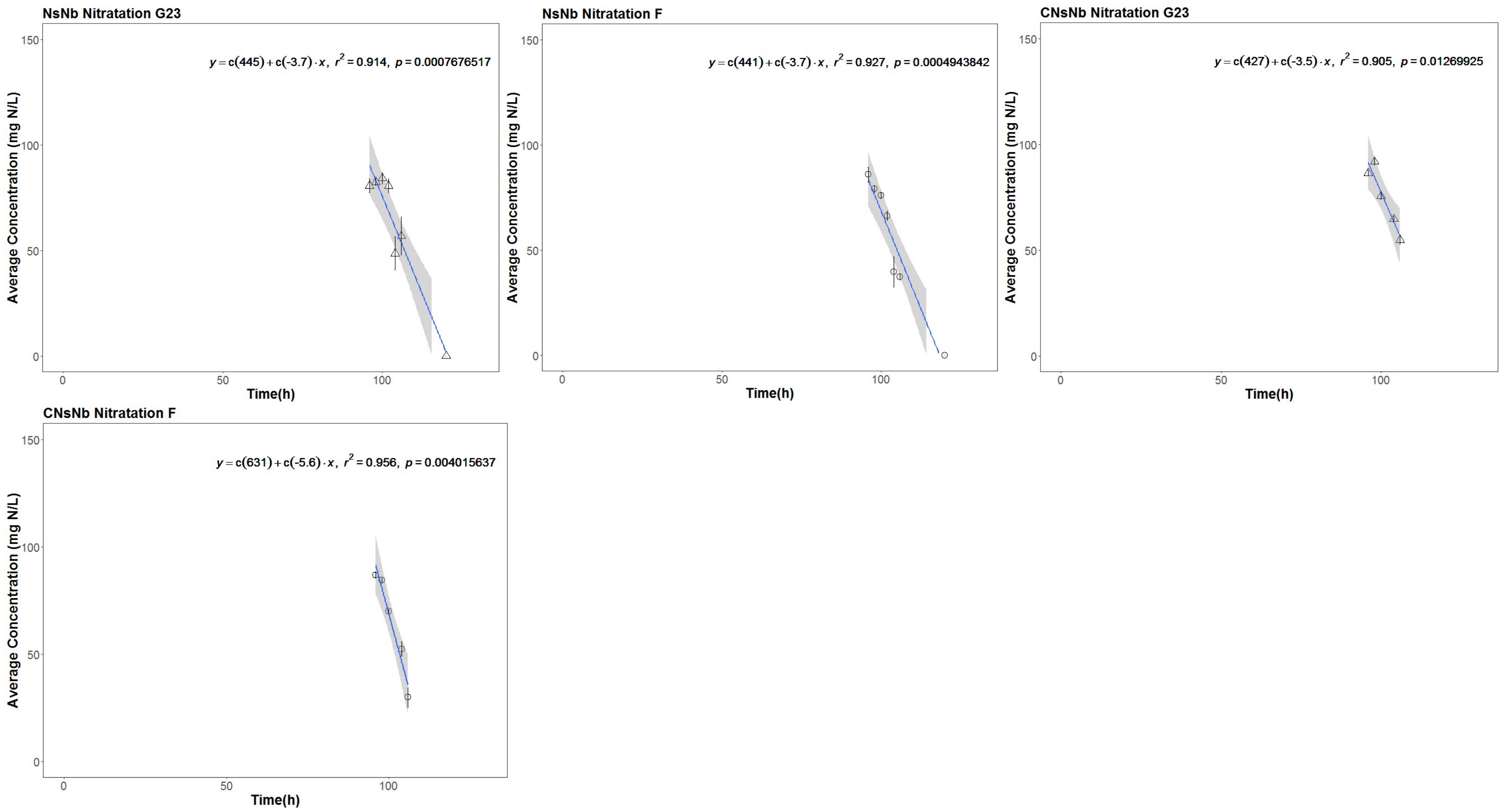


**Figure S5.1: Overview of average concentrations of ammonium and nitrite (mg N $L^{-1}$) in sixplicate for F (o) and G23 ($\Delta$) experiments on the axenic cultures and defined communities. The plotted regression line indicates the volumetric activity per hour.**

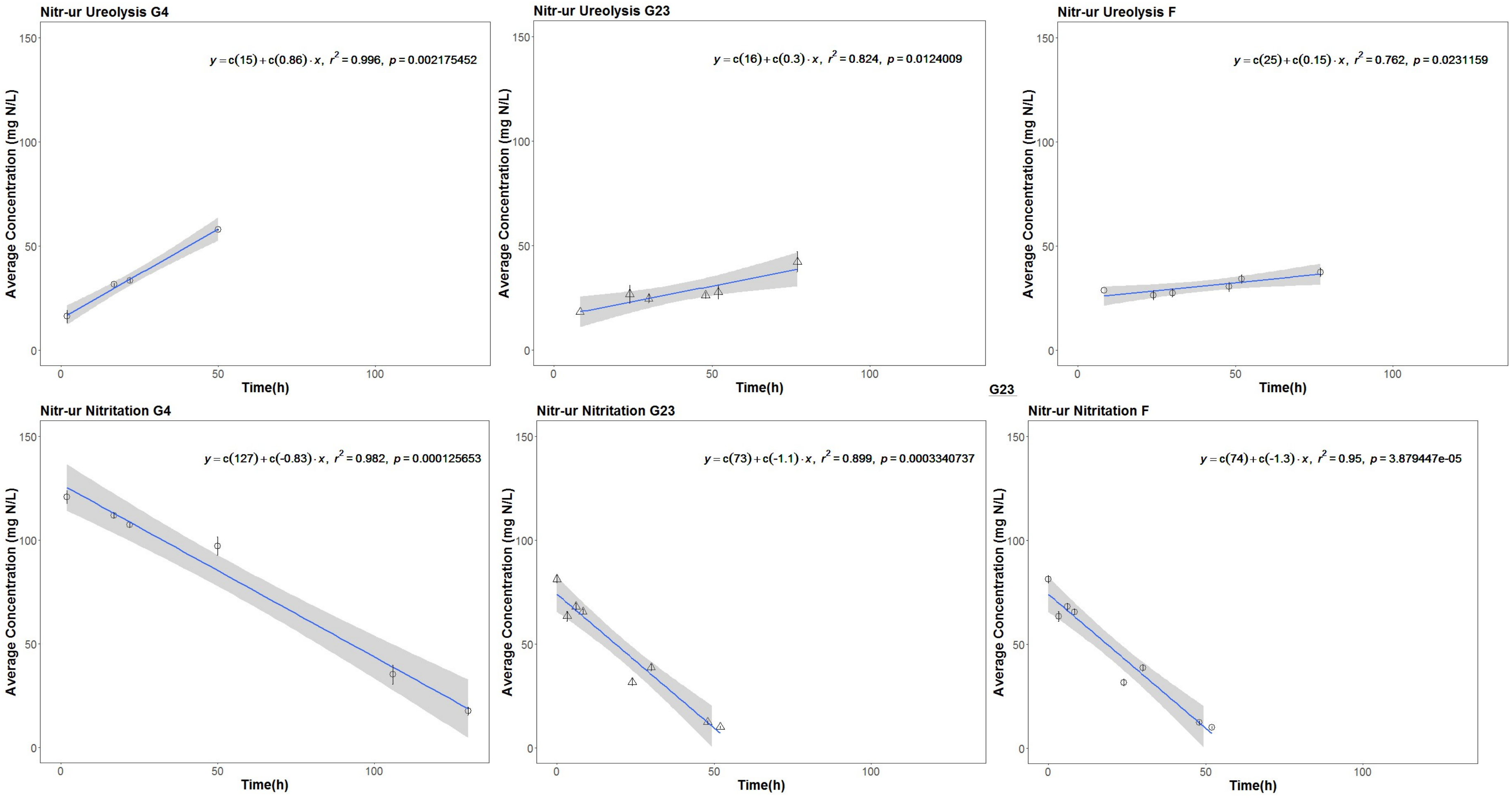
Nitr-ur Ureolysis G4
y = c(15) + c(0.86) · x, r2 = 0.996, p = 0.002175452
Nitr-ur Ureolysis G23
y = c(16) + c(0.3) · x, r2 = 0.824, p = 0.0124009
Nitr-ur Ureolysis F
y = c(25) + c(0.15) · x, r2 = 0.762, p = 0.0231159
G23
Nitr-ur Nitritation G4
y = c(127) + c(-0.83) · x, r2 = 0.982, p = 0.000125653
Nitr-ur Nitritation G23
y = c(73) + c(-1.1) · x, r2 = 0.899, p = 0.0003340737
Nitr-ur Nitritation F
y = c(74) + c(-1.3) · x, r2 = 0.95, p = 3.879447e-05
Average Concentration (mg N/L)
Time(h)

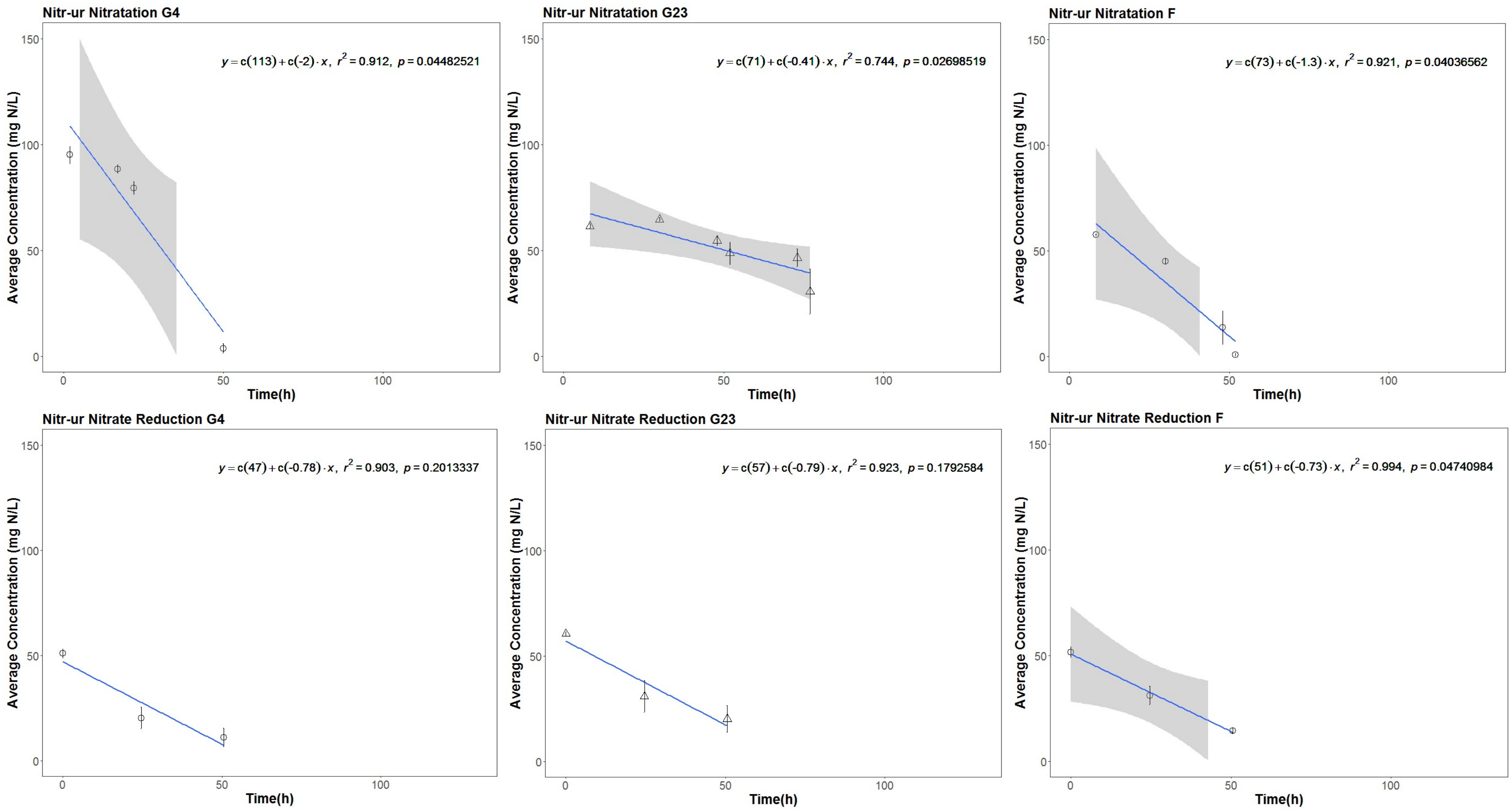
Nitr-ur Nitratation G4
y = c(113) + c(-2) · x, r2 = 0.912, p = 0.04482521
Nitr-ur Nitratation G23
y = c(71) + c(-0.41) · x, r2 = 0.744, p = 0.02698519
Nitr-ur Nitratation F
y = c(73) + c(-1.3) · x, r2 = 0.921, p = 0.04036562
Nitr-ur Nitrate Reduction G4
y = c(47) + c(-0.78) · x, r2 = 0.903, p = 0.2013337
Nitr-ur Nitrate Reduction G23
y = c(57) + c(-0.79) · x, r2 = 0.923, p = 0.1792584
Nitr-ur Nitrate Reduction F
y = c(51) + c(-0.73) · x, r2 = 0.994, p = 0.04740984
Average Concentration (mg N/L)
Time(h)
0
50
100
150

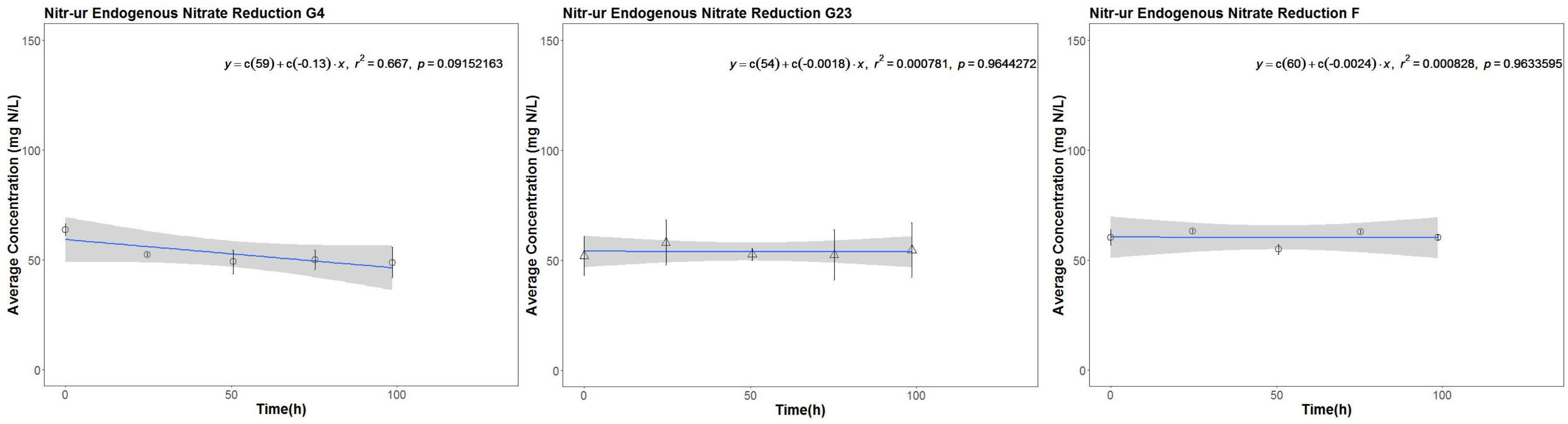


**Figure S5.2: Overview of average concentrations of ammonium, nitrite and nitrate (mg N $L^{-1}$) in sixplicate for G23 and F experiments on the reactor community Nitr-ur and quadruplicate for G4. The plotted regression line indicates the volumetric activity per hour and the 95% confidence interval. Denitrification was measured by nitrate reduction only.**

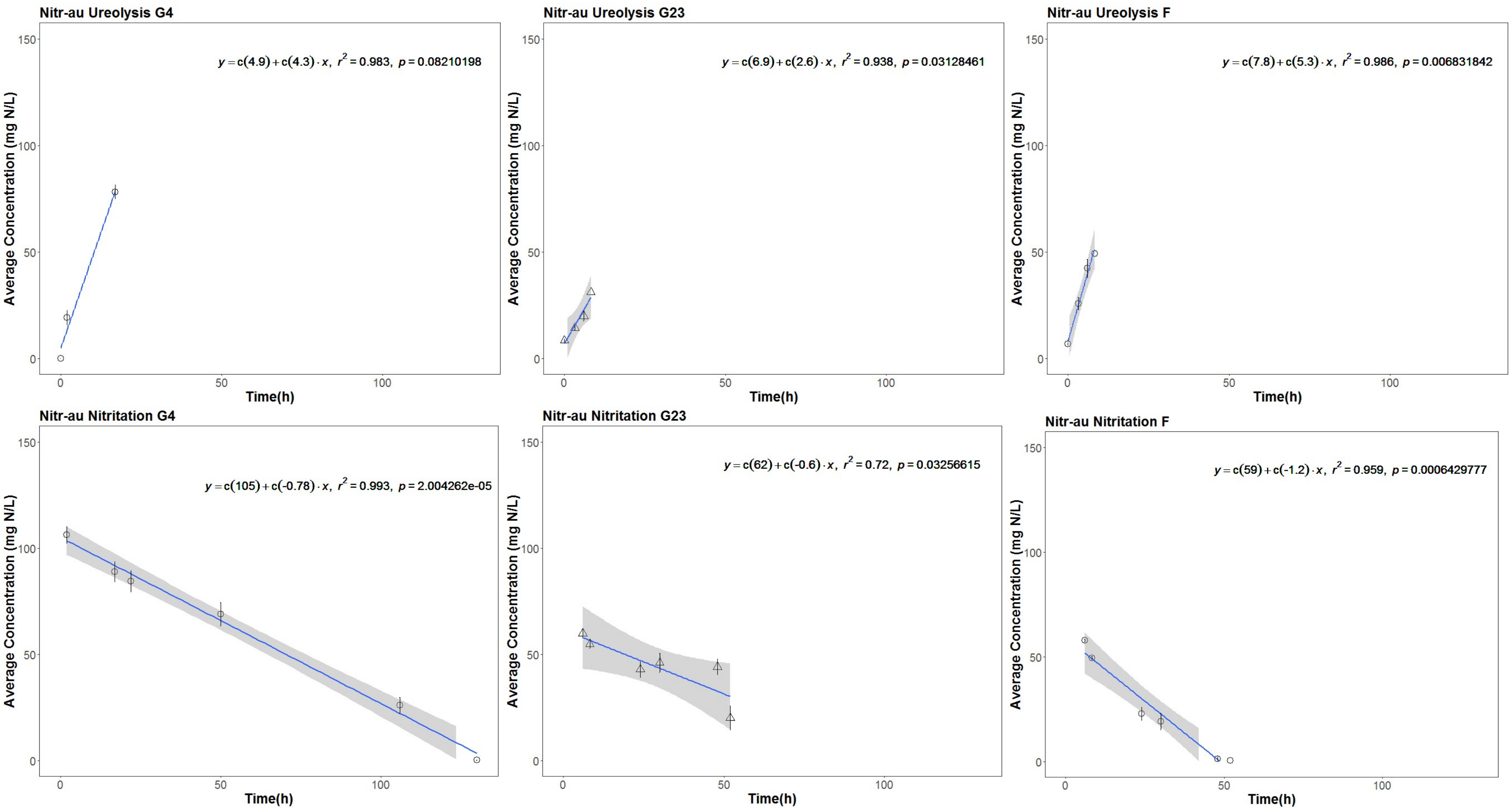
Nitr-au Ureolysis G4
y = c(4.9) + c(4.3) · x, r^2 = 0.983, p = 0.08210198
Nitr-au Ureolysis G23
y = c(6.9) + c(2.6) · x, r^2 = 0.938, p = 0.03128461
Nitr-au Ureolysis F
y = c(7.8) + c(5.3) · x, r^2 = 0.986, p = 0.006831842
Nitr-au Nitritation G4
y = c(105) + c(-0.78) · x, r^2 = 0.993, p = 2.004262e-05
Nitr-au Nitritation G23
y = c(62) + c(-0.6) · x, r^2 = 0.72, p = 0.03256615
Nitr-au Nitritation F
y = c(59) + c(-1.2) · x, r^2 = 0.959, p = 0.0006429777
Average Concentration (mg N/L)
Time(h)
0
50
100
150

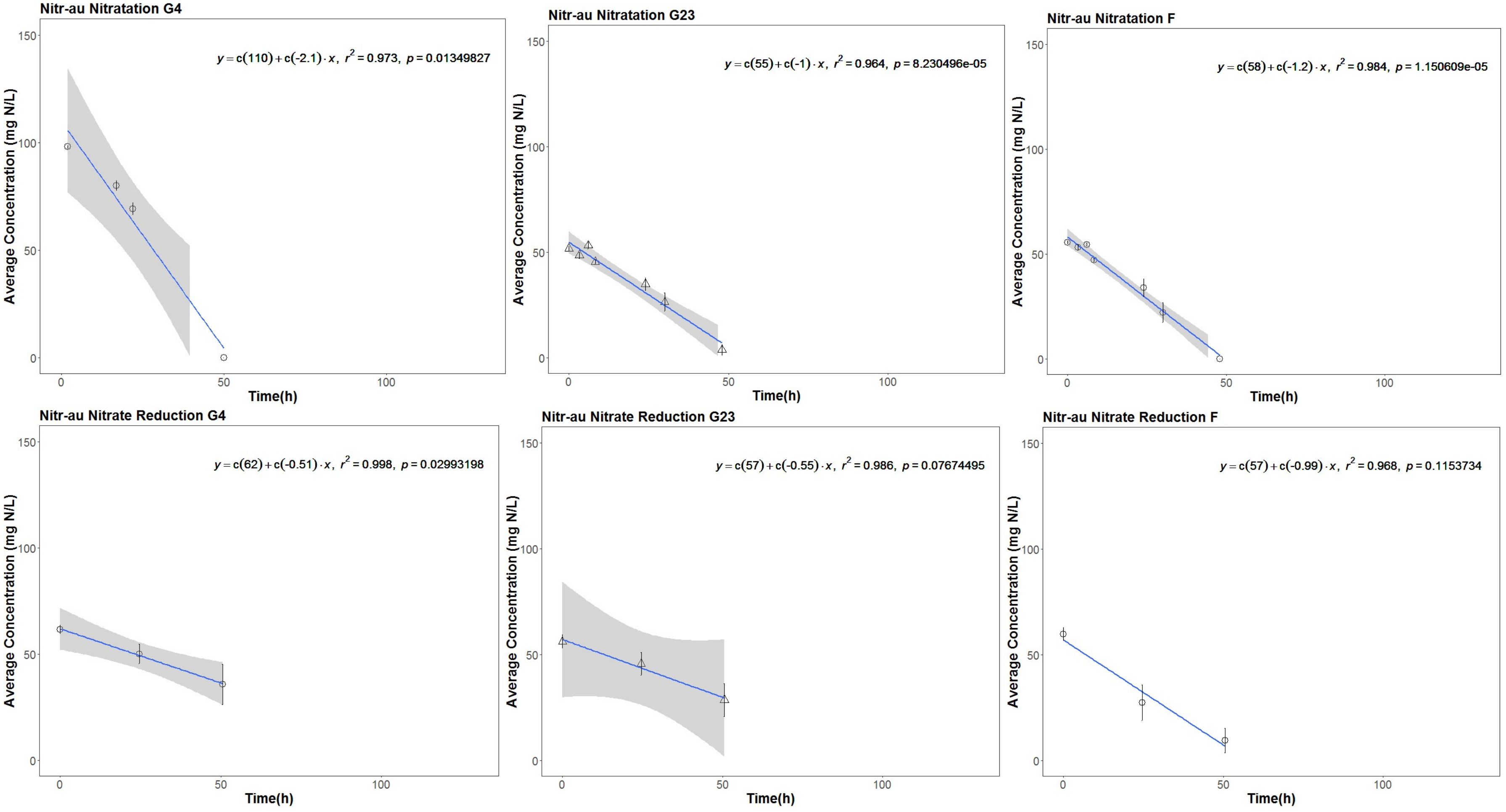

Nitr-au Nitratation G4
y = c(110) + c(-2.1) · x, r^2 = 0.973, p = 0.01349827
Nitr-au Nitratation G23
y = c(55) + c(-1) · x, r^2 = 0.964, p = 8.230496e-05
Nitr-au Nitratation F
y = c(58) + c(-1.2) · x, r^2 = 0.984, p = 1.150609e-05
Nitr-au Nitrate Reduction G4
y = c(62) + c(-0.51) · x, r^2 = 0.998, p = 0.02993198
Nitr-au Nitrate Reduction G23
y = c(57) + c(-0.55) · x, r^2 = 0.986, p = 0.07674495
Nitr-au Nitrate Reduction F
y = c(57) + c(-0.99) · x, r^2 = 0.968, p = 0.1153734
Average Concentration (mg N/L)
Time(h)
0
50
100
150

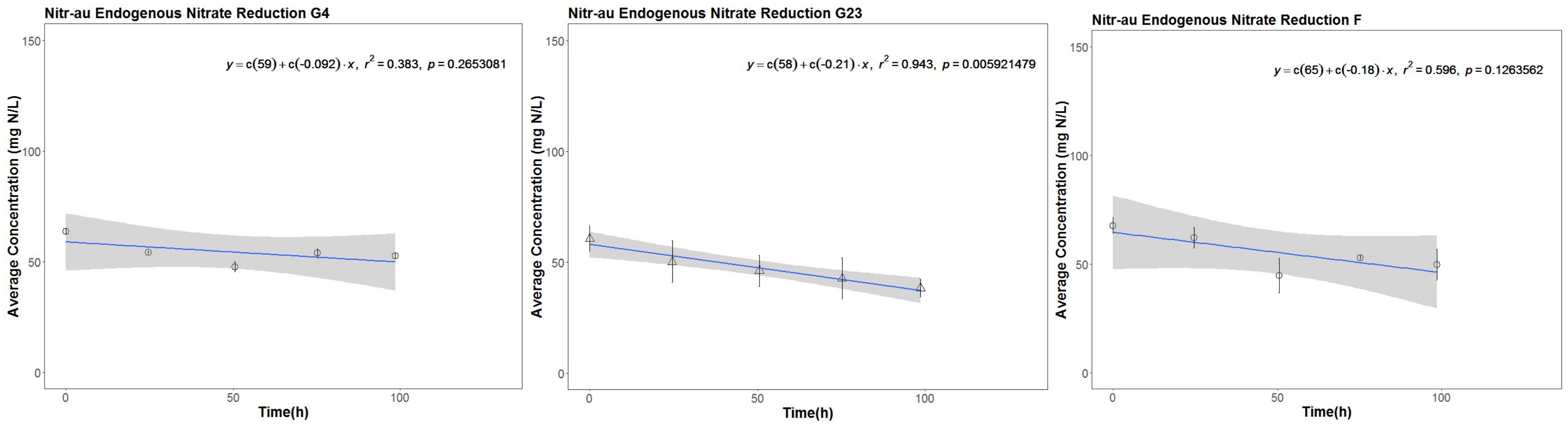


**Figure S5.3: Overview of average concentrations of ammonium, nitrite and nitrate (mg N $L^{-1}$) in sixplicate for G23 and F experiments on the natural community Nitr-au and quadruplicate for G4. The plotted regression line indicates the volumetric activity per hour and the 95% confidence interval. Denitrification was measured by nitrate conversion only.**

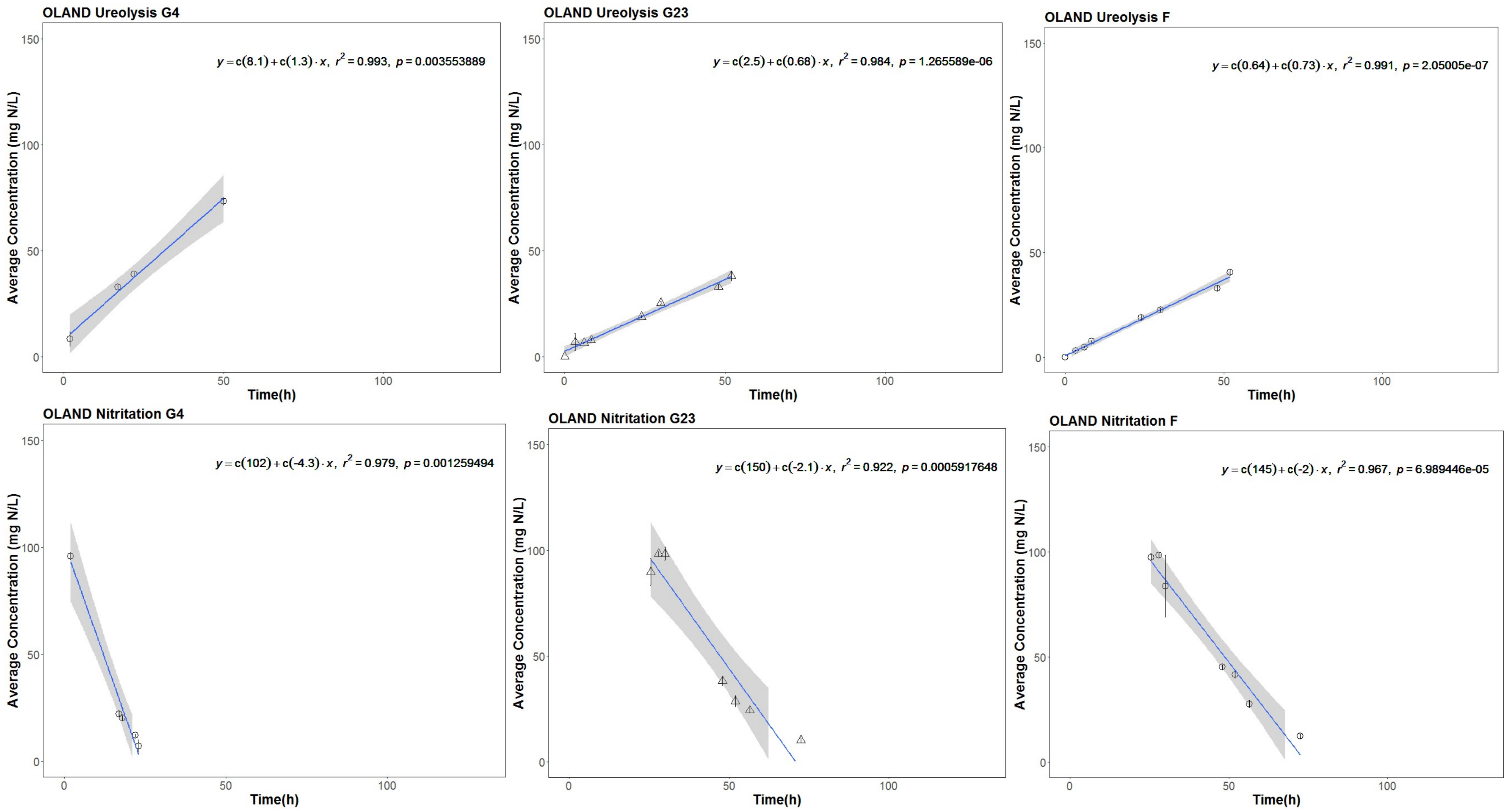
OLAND Ureolysis G4
y = c(8.1) + c(1.3) · x, r2 = 0.993, p = 0.003553889
OLAND Ureolysis G23
y = c(2.5) + c(0.68) · x, r2 = 0.984, p = 1.265589e-06
OLAND Ureolysis F
y = c(0.64) + c(0.73) · x, r2 = 0.991, p = 2.05005e-07
OLAND Nitritation G4
y = c(102) + c(-4.3) · x, r2 = 0.979, p = 0.001259494
OLAND Nitritation G23
y = c(150) + c(-2.1) · x, r2 = 0.922, p = 0.0005917648
OLAND Nitritation F
y = c(145) + c(-2) · x, r2 = 0.967, p = 6.989446e-05
Average Concentration (mg N/L)
Time(h)
0
50
100
150

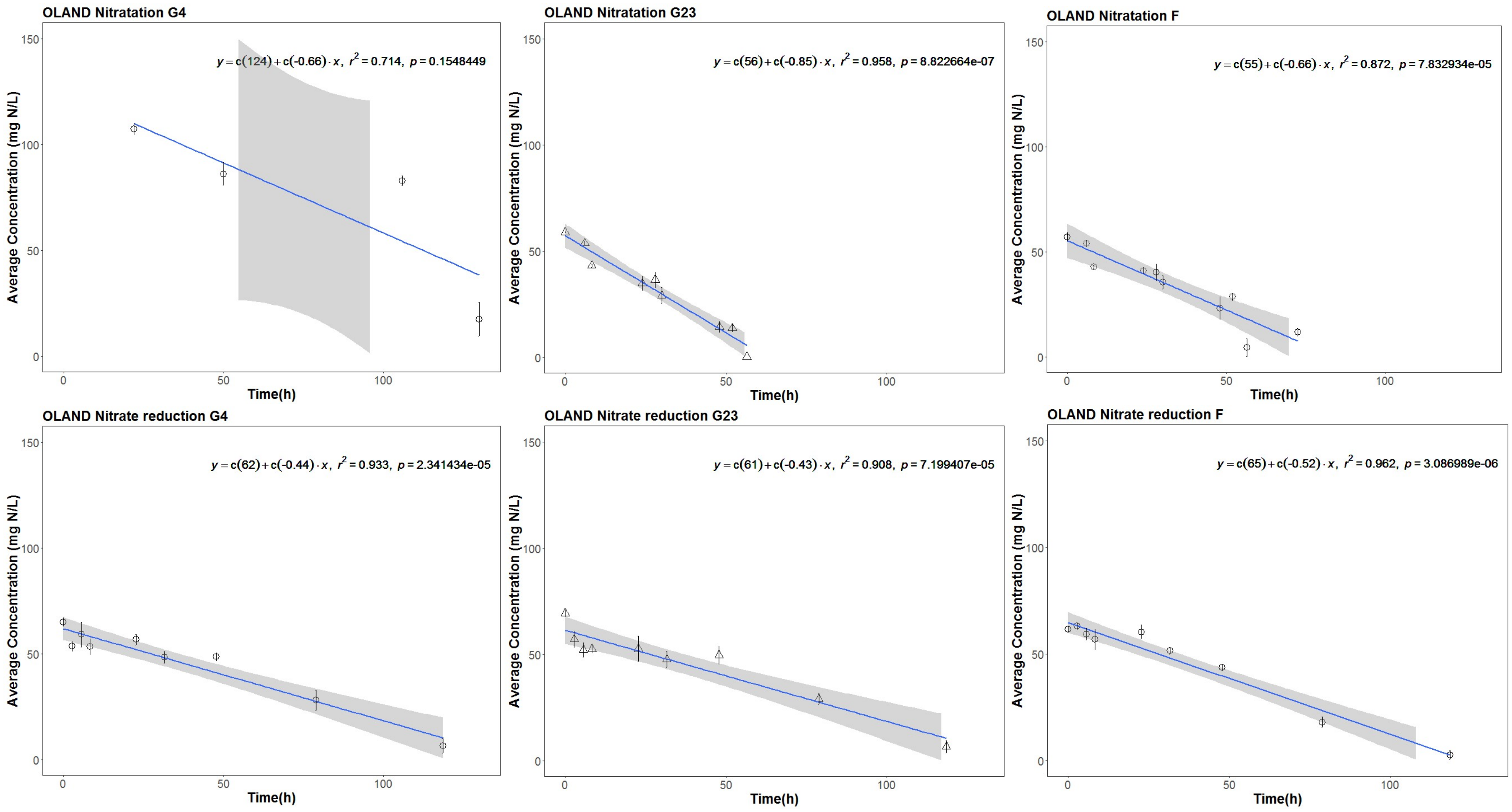

OLAND Nitratation G4
$y = c(124) + c(-0.66) \cdot x,\ r^2 = 0.714,\ p = 0.1548449$
OLAND Nitratation G23
$y = c(56) + c(-0.85) \cdot x,\ r^2 = 0.958,\ p = 8.822664e-07$
OLAND Nitratation F
$y = c(55) + c(-0.66) \cdot x,\ r^2 = 0.872,\ p = 7.832934e-05$
OLAND Nitrate reduction G4
$y = c(62) + c(-0.44) \cdot x,\ r^2 = 0.933,\ p = 2.341434e-05$
OLAND Nitrate reduction G23
$y = c(61) + c(-0.43) \cdot x,\ r^2 = 0.908,\ p = 7.199407e-05$
OLAND Nitrate reduction F
$y = c(65) + c(-0.52) \cdot x,\ r^2 = 0.962,\ p = 3.086989e-06$
Average Concentration (mg N/L)
Time(h)

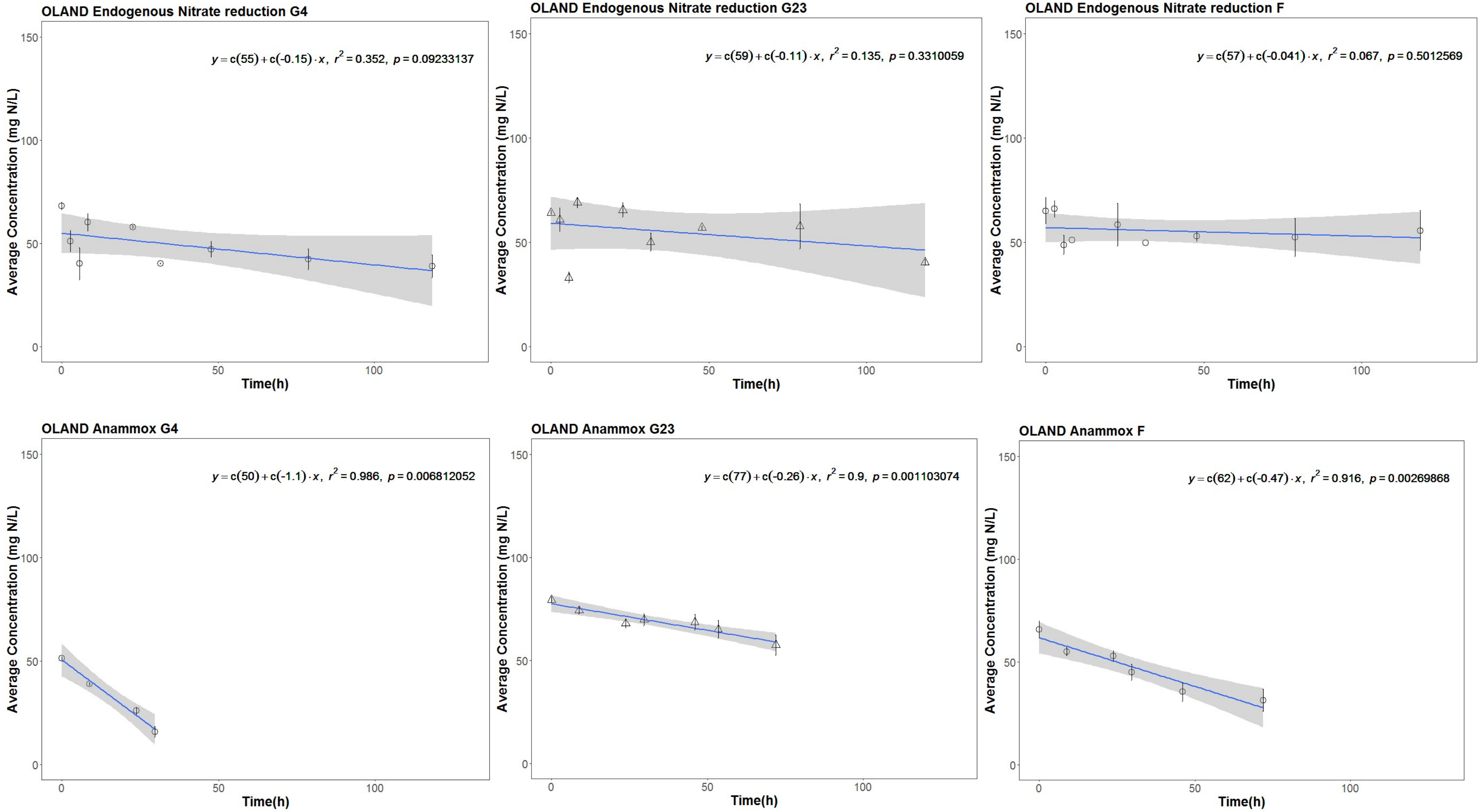


**Figure S5.4: Overview of average concentrations of ammonium, nitrite and nitrate (mg N L-1) in sixplicate for G23 and F experiments on the natural community OLAND and quadruplicate for G4. The plotted regression line indicates the volumetric activity per hour and the 95% confidence interval. Denitrification was measured by nitrate conversion only.**
